# MERTK coordinates efferocytosis by regulating integrin localization and activation

**DOI:** 10.1242/jcs.264792

**Published:** 2026-03-25

**Authors:** Brandon H. Dickson, Tarannum Tasnim, Rachel A. Nicholson, Natalie Stanlake, Austin L. Lam, Angela M. Vrieze, Eoin N. Blythe, Gregory A. Dekaban, Bryan Heit

**Affiliations:** Department of Microbiology and Immunology, and the Western Infection, Immunity and Inflammation Centre, The University of Western Ontario, London, Ontario, N6A 5C1, Canada

**Keywords:** Efferocytosis, MERTK, Integrin, Signaling, Macrophages

## Abstract

Efferocytosis is mediated by MERTK in many tissues, but the signaling pathway and molecular mechanisms used by MERTK to engulf apoptotic cells is largely unknown. Here, using mass spectrometry and super-resolution microscopy, we identified 180 nm receptor complexes comprised of MERTK, β_2_ integrins and several associated signaling molecules. Efferocytosis was found to be dependent on both MERTK and β_2_ integrins, with MERTK inducing the conformational change of β_2_ integrins from low to high-affinity via a PI3K-dependent pathway, with the active integrins then mediating the expansion of an efferocytic synapse around the apoptotic cell. This synapse was highly structured, with MERTK retained by ligand-induced clustering in the synapse centre, while β_2_ integrins and actin form a Src family kinase and FAK-dependent expanding ring that defined the leading edge of the efferocytic synapse. These findings provide new insights into the function of this crucial homeostatic receptor and provide new insights into how MERTK mutations and signaling defects might contribute to inflammatory and autoimmune diseases.

## Teaser

**Summary:** MERTK mediates apoptotic cell engulfment through forming a structured synapse comprising a bounding ring of actin and activated integrins, and a MERTK-enriched central core.

## INTRODUCTION

The great diversity of phagocytic receptors is a key component of the ability of the immune system to clear pathogens, as this diversity allows phagocytes to engage a broad range of pathogens based on unique pathogen surface chemistry (e.g. dectin-1 binding of fungal glucans), via cooperation with other pathogen-detection systems (e.g. complement-mediated phagocytosis), and to utilize the vast repertoire of the adaptive immune system (e.g. Fc-receptor-mediated phagocytosis) (Brown and Gordon, 2001; Bruhns et al., 2009; Xu et al., 2017). Like phagocytosis, efferocytosis – the phagocytic removal of apoptotic cells – is driven by an expansive repertoire of receptors. But unlike phagocytosis, the purpose of this diversity is unclear. Indeed, with only a few exceptions, efferocytic receptors recognize the same ligand – phosphatidylserine (PtdSer; reviewed in Lam and Heit, 2021). This lipid ‘eat me’ signal is normally restricted to the inner leaflet of the plasma membrane, but during apoptosis, caspases activate the scramblase XKR8 and inactivate the flippases ATP11A and ATP11C, thus exposing PtdSer on the outer leaflet (Segawa et al., 2014; Suzuki et al., 2016). Although some of this receptor diversity might be explained by cell-type specific expression of some receptors, most efferocytes express multiple efferocytic receptors (Lam and Heit, 2021). For example, macrophages express MERTK, Axl, Tyro3, TIM-4 (also known as TIMD4), α_x_β_2_ integrin (subunits also known as CD11c and CD18), multiple β_5_ integrins, BAI-1, LRP-1 and CD36 – all receptors reported to bind to apoptotic cells either directly or via opsonins (Bain et al., 2013; Gardai et al., 2005; Heit et al., 2013; Nishi et al., 2014; Sándor et al., 2016; Seitz et al., 2007). Why a single cell type would need to express such a large repertoire of receptors that recognize the same ligand, especially given that apoptotic cells expose abundant quantities of PtdSer – upwards of 10% of total outer leaflet lipid, remains unclear (Leventis and Grinstein, 2010). One possible explanation for this diversity is the need to closely regulate ‘multi-purpose’ receptors, such as integrins. Indeed, both MERTK and TIM-4 have been reported to require integrins for efficient apoptotic cell uptake, relying on the opsonins MFG-E8 and soluble CD93 (sCD93) to bridge integrins to the apoptotic cell (Blackburn et al., 2019; Finnemann and Nandrot, 2006; Flannagan et al., 2014). These same integrins, when provided different opsonins or ligands, can also serve as phagocytic receptors, anchors to the extracellular matrix, mediators of intravascular adhesion and as adhesion receptors for cell migration. Some integrins even serve all of these roles – α_x_β_2_ mediates efferocytosis via the opsonin sCD93, pathogen phagocytosis via the opsonin iC3b, intravascular adhesion via binding to ICAM-2 and VCAM-1, and mediates cell migration (Blackburn et al., 2019; Lukácsi et al., 2020; Nagy-Baló et al., 2020; Sadhu et al., 2007). The use of integrins for this broad array of activities likely evolved to take advantage of features not found in other adhesion receptors – the ability to regulate integrin affinity via conformational changes, the high affinity of integrins for their ligands, and the ability of integrins to engage actin-based force-generating machinery within the cell (Jaumouillé et al., 2019; Lefort et al., 2009; McDowall et al., 1998).

This diverse range of activities requires that integrins be carefully regulated. This regulation generally occurs via other receptors that modulate integrin affinity through inside-out signaling, allowing these receptors to direct the resulting pattern of integrin activation. Antibody-mediated phagocytosis via Fcγ receptors (FcγR) shares many mechanical similarities to efferocytosis and might provide insights into how integrins are regulated during efferocytosis. During the phagocytosis of IgG-opsonized targets, FcγR signaling induces the formation of a phagocytic synapse in which FcγRs are concentrated in the center and are surrounded by a bounding ring of active α_m_β_2_ integrin (subunits also known as CD11b and CD18, and together as Mac-1) (Freeman et al., 2016). This outer ring of α_m_β_2_ does not appear to serve an adhesive or force-generating role, and instead forms a diffusion barrier that excludes the phosphatase CD45 (also known as PTPRC). This exclusion of CD45 from the phagocytic cup ensures uninterrupted Src-family kinase (SFK) signaling, which regulates the contractile forces required for engulfment of the antibody-opsonized pathogen (Choraghe et al., 2020). In contrast, during complement-mediated phagocytosis, α_m_β_2_ and α_x_β_2_ bind to complement iC3b, which is deposited on the surface of pathogens following activation of the complement cascade (Bilsland et al., 1994; Xu et al., 2017). Unlike in FcγR-mediated efferocytosis, the integrins are the primary phagocytic receptor in this form of phagocytosis, providing both adhesion to the target and generating the actin-mediated contractile force that draws the pathogen into the phagocyte (Jensen et al., 2021; Torres-Gomez et al., 2020). Clearly, the roles of integrins in phagocytosis-like processes can vary depending on the ligands present on the phagocytic target, and on the signaling being generated by other receptors within or near the phagocytic synapse.

MERTK is the sole or predominant efferocytic receptor in multiple tissues including the eye, brain and heart, and is highly expressed in macrophages – cells which function as the primary efferocyte in many tissues (DeBerge et al., 2017; Finnemann and Nandrot, 2006; Seitz et al., 2007). Single-nucleotide polymorphisms (SNPs) in MERTK and its opsonins are associated with a range of diseases including retinitis pigmentosa, male infertility, autoimmune disorders, such as multiple sclerosis and systemic lupus erythematosus, neurological disorders, including Alzheimer's disease, and inflammatory diseases including atherosclerosis (Binder et al., 2016; Brea-Fernández et al., 2008; Chen et al., 2009; Ma et al., 2011). MERTK is bridged to PtdSer on apoptotic cells by two well-characterized opsonins – Gas6 and Protein S (also known as PROS). Engagement of an apoptotic cell activates the intrinsic tyrosine kinase domain of MERTK, initiating a signaling pathway whose membrane-proximal signaling is incompletely understood, but which converges on the adaptor Grb2, phosphoinositide 3-kinase (PI3K), SFKs and Vav3 (Shelby et al., 2013). In retinal pigment epithelial cells, this pathway engages α_v_β_5_ integrin to mediate the efferocytosis of shed photoreceptor outer segments, with α_v_β_5_ proposed to activate Rac1, which in turn mediates the actin reorganization necessary to engulf the apoptotic cell (Mao and Finnemann, 2012; Nandrot et al., 2012; Wu et al., 2005). However, the role of integrins in MERTK-mediated efferocytosis is controversial, with some studies suggesting that integrin binding is a pre-requisite for MERTK activation, others suggesting that MERTK activates integrins and yet other studies suggesting that integrins are dispensable for MERTK-mediated phagocytosis (Dransfield et al., 2015; Finnemann and Nandrot, 2006; Hu et al., 2004; Toda et al., 2014).

Using mass spectrometry (MS), selective engagement of MERTK and β_2_ integrins and quantitative microscopy, we demonstrate that MERTK signaling coordinates the formation a highly ordered synapse-like structure between macrophages and apoptotic cells, with MERTK regulating synapse structure and the positioning and activation of β_2_ integrins within the synapse. This coordinated signaling produces an expanding integrin ring that engulfs the apoptotic cell, with efficient macrophage efferocytosis requiring this coordinated activity of MERTK and β_2_ integrins.

## RESULTS

### Macrophage efferocytic receptor expression and usage

**Fig. 1.**
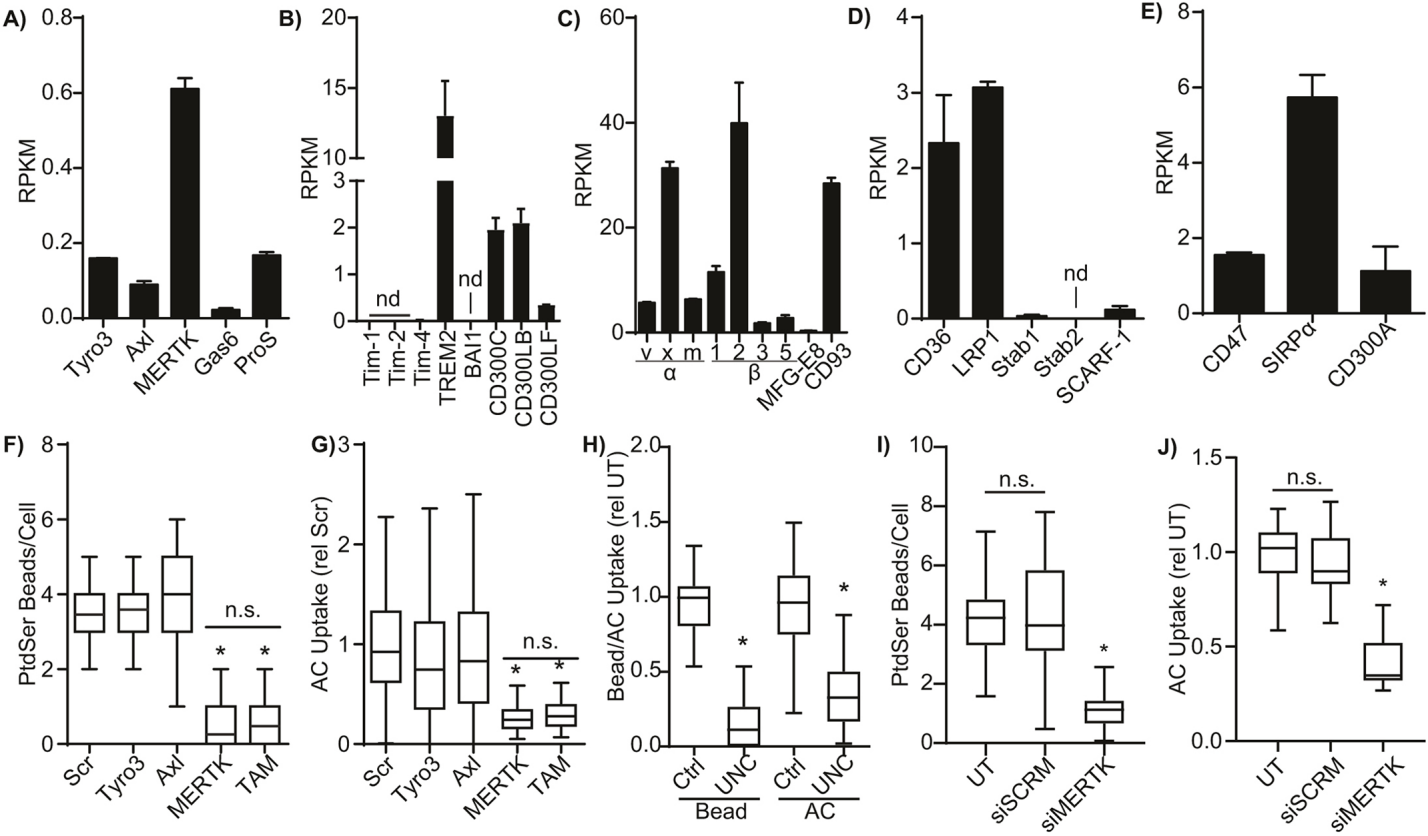
**Efferocytic receptor expression and function in THP1 macrophages.** (A–E) mRNA expression of (A) TAM-family efferocytic receptors (Tyro3, Axl and MERTK) and their opsonins Gas6 and Protein S (ProS), (B) PtdSer-binding efferocytic receptors, (C) integrin α-chains (α_v_, α_x_, α_m_), integrin β-chains (β_1_, β_2_, β_3_, β_5_) and the opsonins (MFG-E8 and CD93) of efferocytic integrins, (D) scavenger receptors known to bind to apoptotic cells, and (E) inhibitory efferocytic receptors as quantified from previously published RNA-seq data from PMA-differentiated THP1 macrophages. nd, not detected (Yin et al., 2020; doi:10.5683/SP2/ISOA8W). (F,G) Quantification of the efferocytosis of serum-opsonized apoptotic mimics (F) or apoptotic cells (AC) (G) by THP1 macrophages where Tyro3, Axl, MERTK or all three receptors (TAM) were depleted with siRNA. Scr, scrambled siRNA control. (H) Efferocytic uptake of serum-opsonized apoptotic mimics and apoptotic Jurkat T cells by macrophages treated with 5 µM of the MERTK inhibitor UNC2250 (UNC) or vehicle control (Ctrl). (I,J) Efferocytic uptake of serum-opsonized PtdSer-coated beads (I) or apoptotic Jurkat cells (J) by human peripheral-blood monocytic cell-derived M0 macrophages that were untreated (UT), or treated with either a non-targeting (siSCRM) or MERTK-depleting (siMERTK) cell penetrant siRNA. Data are plotted as mean±s.e.m. (A–E) or as box plots showing median and interquartile range, with whiskers indicating the 5–95th percentiles (F–H). *n*=3. *P<0.05; *n*.s.=*P*>0.05 compared to Scr (F,G) or UT (H) (Kruskal–Wallis test with Dunn's multiple comparisons test).

Phorbol 12-myristate 13-acetate (PMA)-differentiated THP1 macrophages expressed a broad range of efferocytic receptors, including all three members of the Tyro3, Axl and MERTK (TAM) family and their opsonins (Fig. 1A), receptors which directly bind PtdSer (Fig. 1B), integrins (Fig. 1C), scavenger receptors (Fig. 1D) and inhibitory receptors (Fig. 1E). Apoptotic Jurkat cells and apoptotic mimics comprising 3 µm beads coated in a 20:80 mixture of PtdSer:phosphatidylcholine (PtdChol) were opsonized with human serum – which contains apoptotic cell opsonins for integrins (MFG-E8 and complement) and TAM receptors (Gas6 and ProS) – and were used to determine the relative role of the TAM receptors in macrophage efferocytosis (van der Meer et al., 2014). Despite the wide array of efferocytic receptors expressed in these cells, siRNA knockdown of MERTK abrogated efferocytosis by more than 90% for both apoptotic mimics and apoptotic cells, with even a triple-TAM knockdown having no additive effect over MERTK knockdown alone (Fig. 1F,G; Fig. S1A). A similar trend was observed when THP1 macrophages were treated with the MERTK kinase domain inhibitor UNC2250 (Fig. 1H) (Zhang et al., 2013). To confirm that these trends were not a cell line artifact, human peripheral blood mononuclear cells were differentiated into M0-polarized macrophages (PBMC-M0) and MERTK depleted using a cell-penetrant siRNA (Fig. 1I,J), with these cells exhibiting the same dependency on MERTK for efferocytosis as THP1-derived macrophages. Although these results do not eliminate potential roles for the other receptors at later stages of apoptotic cell engulfment, they indicate that at a minimum, macrophages rely on MERTK for the initial recognition of apoptotic cells. Given this central role of MERTK in the initial recognition of apoptotic cells, we further probed MERTK organization on macrophages, using MERTK-selective targets to limit signaling through other efferocytic receptors.

### Characterization of the MERTK signalosome

**Fig. 2.**
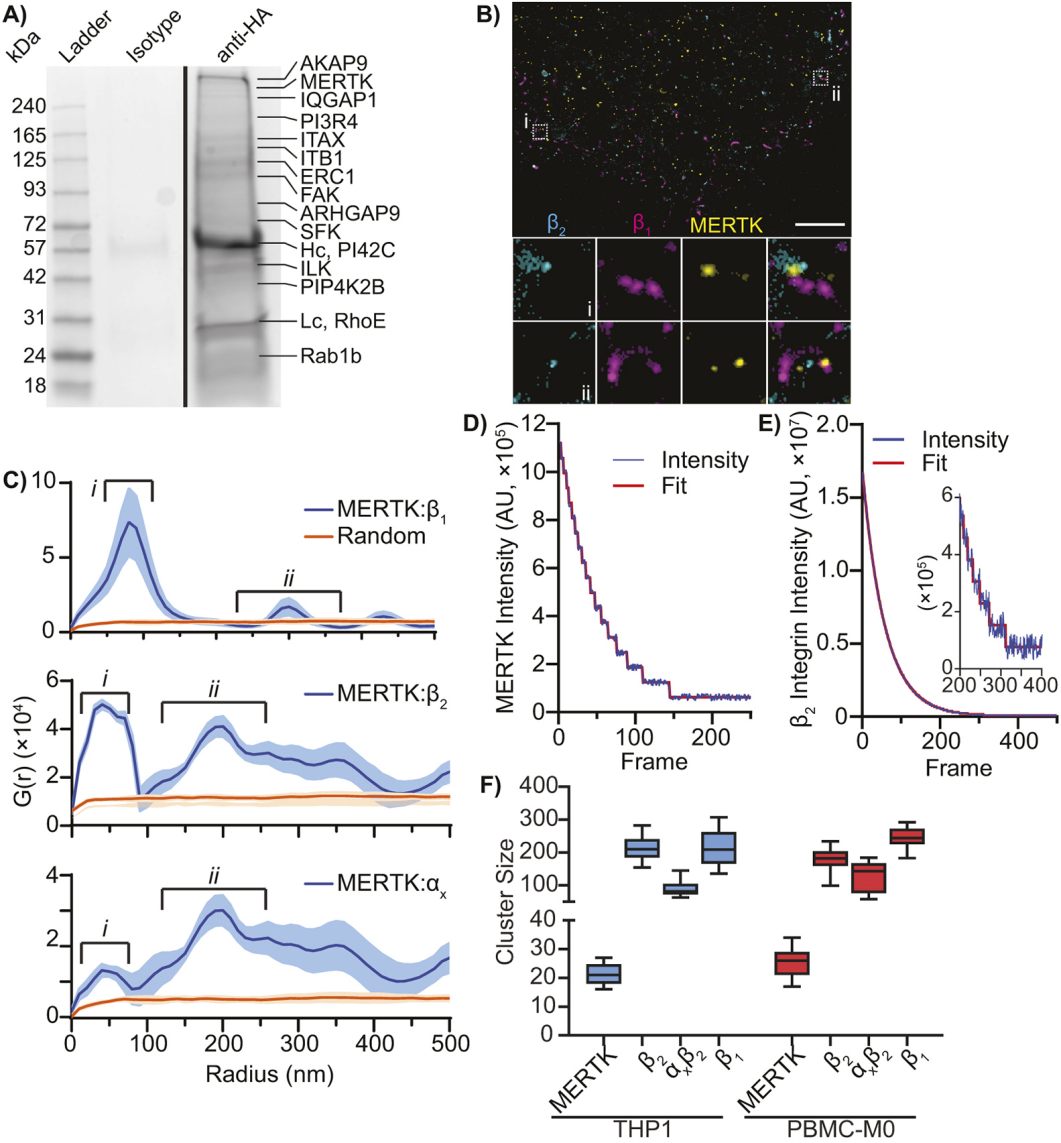
**Identification of MERTK co-receptors.** (A) Representative anti-HA-MERTK immunoprecipitation. Reversible crosslinking and MS were used to identify proteins which co-immunoprecipitate with MERTK. Isotype, irrelevant antigen antibody; anti-HA, HA–MERTK immunoprecipitation. Major proteins found in each band are identified; Hc, heavy chain of precipitating antibody, Lc, light chain of precipitating antibody. Image representative of three repeats. (B) Representative GSDM image of MERTK (yellow) and β_1_ integrin (magenta) and β_2_ integrin (cyan) on the macrophage surface. Magnified views underneath are of the area indicated by two dashed boxes (i and ii) showing MERTK co-clustered with both β_1_ and β_2_ (i) and co-clustered with only β_1_ (ii). Scale bar: 2 µm. (C) Radial distribution analysis of the density of β_1_, β_2_ and α_x_ integrins as a function of their distance from MERTK (blue). The integrins show two distinct clusters with MERTK: (i) co-clustering within the ∼120 nm diameter MERTK clusters, and (ii) clusters which touch, but are not intermixed with, the MERTK clusters. Random (orange), G(r) measured when the position of MERTK has been randomized. Blue shading shows 95% confidence interval. (D–F) Quantification of the number of MERTK and integrins present in MERTK clusters as measured by stepwise photobleaching analysis, showing representative bleaching curves and bleaching-step fits for MERTK (D) and β_2_ integrin (E) on PBMC-M0 macrophages, and (F) quantification of the number of molecules per MERTK cluster on THP1-derived versus PBMC-M0 macrophages. *N*=3–5. Box plots in F show median and interquartile range, with whiskers indicating 5–95th percentiles. No statistically significant differences were observed between the cluster sizes present on THP1-derived versus PBMC-M0 macrophages compared to Randomized (one-way ANOVA with Tukey correction). AU, arbitrary units.

We previously determined that human MERTK is highly clustered within regions ∼180 nm in diameter on the surface of resting human macrophages (Evans et al., 2017), suggesting that MERTK exists in pre-formed complexes prior to efferocytosis. To determine whether other proteins were co-clustered in these MERTK clusters, we expressed MERTK with an extracellular HA tag, and then immunoprecipitated MERTK using a reversible cross-linking approach designed to precipitate membrane proteins with minimal non-specific interactions (Smith et al., 2011). Immunoprecipitation followed by MS identified several proteins associated with MERTK on resting cells, including β_1_ and α_x_β_2_ integrins, integrin-associated signaling molecules [FAK (also known as PTK2) and ILK], members of the PI3K signaling pathway, regulators of Rho-family GTPases and several kinases including multiple SFKs (Fig. 2A; Table 1). As expected, proteins known to be recruited to activated MERTK (e.g. Grb2) were not found in our immunoprecipitates, confirming that we immunoprecipitated MERTK in its inactive state (Shelby et al., 2013). To validate these interactions and investigate the spatial organization of the immunoprecipitated proteins, spatial statistics were applied to ground state depletion microscopy (GSDM) images with ∼20 nm resolution, using Fab fragments to avoid artifactual cross-linking or clustering of the labeled proteins (Caetano et al., 2015). As is common with plasma membrane proteins, most of the detected proteins exhibited significant self-clustering (Fig. 2B; Fig. S2A–D), with the bulk of MERTK clustered into structures ∼180 nm in diameter, similar to what we reported previously (Evans et al., 2017). Radial distribution analysis identified an unexpectedly complex spatial relationship between MERTK and the β_1_, β_2_ and α_x_β_2_ integrins (Fig. 2C). β_1_ integrin was strongly enriched at distances from 20 to 100 nm from MERTK, indicating that MERTK and β_1_ integrin are co-clustered in regions 100–200 nm in diameter. Although a portion of β_2_ and α_x_β_2_ integrins co-clustered with MERTK in regions of similar diameter, a secondary enrichment of β_2_ and α_x_β_2_ integrins was found at radii of 100 nm to 300 nm – indicating that in addition to being co-clustered with MERTK, that clusters of these integrins also contact MERTK clusters without intermixing (Fig. 2C; Fig. S2A). MERTK was present at relatively low abundance in both the immunoprecipitates (Fig. 2A) and in the integrin clusters observed in the GSDM images (Fig. 2B–E; Fig. S2A–D), consistent with our previous observation that human MERTK has evolved recently to be expressed at lower levels than in other apes (Evans et al., 2017). To quantify the stoichiometry of MERTK versus the integrins, we used stepwise photobleaching to count the number of MERTK and integrins present in these clusters (Hummert et al., 2021). Using this approach on THP1-derived and PBMC-M0 macrophages, we found that MERTK was present in small clusters containing fewer than 30 MERTK/cluster (Fig. 2D,F). The integrin clusters were too large to directly analyze with this approach (Fig. 2E), but by completely photobleaching these complexes and performing a linear regression on the final 10–15 photobleaching events, we were able to calculate the integrated intensity of single integrins in our images (Fig. S2E), and from there estimated the number of integrins present in the MERTK-colocalizing integrin complexes (Fig. 2E,F). We estimated that the β_2_ integrin clusters contain between 120 and 290 molecules, with about a third of these being α_x_β_2_. MERTK-associated β_1_ integrin clusters were of a similar size to the β_2_ integrin clusters (Fig. 2F). This clustering of MERTK, and its inclusion into larger multi-protein complexes, potentially overcomes the impact of low MERTK expression via increased avidity of MERTK–ligand interactions and providing greater access to its integrin co-receptors.

**Table 1.** Proteins identified by MS following MERTK immunoprecipitation

Gene symbol	Peptides	Coverage (%)	*P-*value
Integrins			
*ITB1* (β_1_ integrin)	15	21	9.43×10^−4^
*ITAX* (α_x_ integrin)	55	22	5.47×10^−4^
*ITB2* (β_2_ integrin)	31	19	2.76×10^−4^
*ILK*	6	8	1.19×10^−3^
*PTK2* (FAK)	3	3	1.44×10^−2^
GTPases			
*IQGAP1*	18	31	5.49×10^−4^
*RhoE*	6	2	2.66×10^−3^
*ARHGAP9*	4	5	2.04×10^−3^
Src-family kinases			
* Lyn*	2	2	3.51×10^−2^
* Fgr*	3	2	2.63×10^−2^
* Hck*	2	3	3.42×10^−2^
* Fyn*	4	3	1.78×10^−2^
Phosphatidylinositol signaling			
* PI3R4*	4	4	1.14×10^−3^
* PIP4K2B*	3	1	2.99×10^−2^
* PI42C*	8	3	1.34×10^−3^
Protein kinases			
* Erc1*	8	7	1.47×10^−3^
* MAP4K4*	5	5	1.06×10^−3^
* RASA2*	2	1	4.31×10^−2^
* AKAP9*	2	3	1.87×10^−2^

*P*-value assessed by a linear discriminative function (LDF) test. Data from three replicates.

**Fig. 3.**
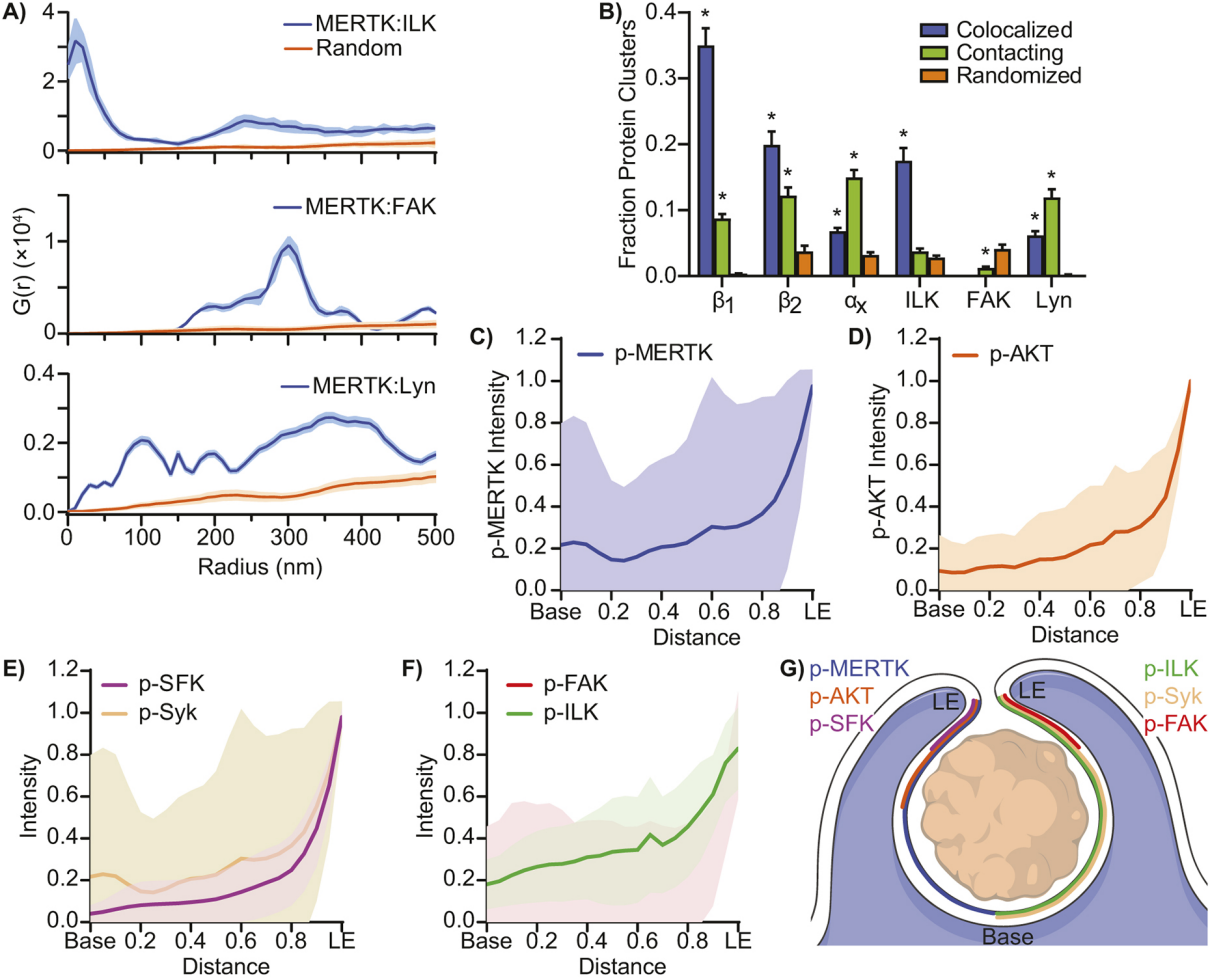
**Characterization of MERTK-associated signaling molecules.** (A) Radial distribution analysis of the co-clustering of ILK, FAK and Lyn with MERTK. Random (orange), G(r) measured when the position of MERTK has been randomized. (B) Fraction of protein clusters that are intermixed with MERTK clusters (colocalized) or contacting but not intermixed with MERTK clusters (contacting). Randomized, fraction of clusters which contact or overlap with MERTK when the position of MERTK clusters is randomized. All clusters were identified in GSDM images using the OPTICS algorithm. (C–F) Quantification of the normalized density of phosphorylated (p-)MERTK (C), AKT (D), Src-family kinases (SFK) and their downstream effector Syk (E), and the integrin regulating FAK and ILK (F). B is mean±s.e.m.; all other data is plotted as the median integrated fluorescence intensity and 95% c.i. (shaded area), quantified from the base of the cup to the leading edge (LE). (G) Graphic depicting the approximate localization of the active forms of MERTK (blue), AKT (orange), SFK (purple), ILK (green), Syk, (brown), and FAK (red) within the efferocytic cup. *n*=3–5, *P<0.05 compared to randomized (one-way ANOVA with Tukey correction).

If this model is correct, co-clustering between MERTK and downstream signaling molecules might also occur, to ensure that low-abundance MERTK can readily access its intracellular signaling cascade upon ligation. To assess this possibility, we repeated the radial distribution analysis, looking at the association between MERTK and ILK, Lyn and FAK in THP1 macrophages. ILK and Lyn strongly co-clustered with MERTK in a pattern reminiscent of the integrins (Fig. 3A; Fig. S2B,C). Unexpectedly, FAK was excluded from MERTK clusters, and instead was enriched in regions neighboring MERTK, suggesting that FAK likely co-precipitated with MERTK-associated integrins rather than with MERTK itself (Fig. 3A; Fig. S2D). To ensure that these were bona fide interactions, and not merely coincidental interactions creating the illusion of co-clustering, the positions of all molecules in the image were randomized over the same area and the co-clustering analysis repeated, with this analysis demonstrating that these co-clusters and contacting clusters occurred with a frequency 50 to 2000 times that expected of randomly distributed proteins (Fig. 3A). To further characterize the interaction of MERTK with these proteins, individual clusters of individual proteins were identified by OPTICS segmentation (Caetano et al., 2015) and the fraction of each protein which overlapped with a cluster of MERTK, or physically contacting but not intermixed with MERTK, determined (Fig. 3B). Consistent with the radial distribution analysis, all three integrins interacted extensively with MERTK, as did ILK and Lyn. These interactions were not due to chance positioning of protein clusters, as the observed degree of overlapping and contacting clusters was greatly decreased when the position of MERTK clusters were randomized over an equivalent area (Fig. 3B). Nor were these interactions artefacts caused by membrane ruffles or other forms of membrane enrichment (Fig. S2F).

We next determined where active (phosphorylated) MERTK, ILK, FAK, SFK, the downstream SFK effector Syk and AKT1 (a proxy for PI3K signaling) were localized in the efferocytic cups of macrophages engulfing 5 μm apoptotic cell mimics opsonized with Gas6 and MFG-E8. Membrane staining revealed that the macrophages extended a thin cup-like structure around the targets, similar to those observed around phagocytic targets (Fig. S2G–L). Immunostaining with activation-specific antibodies revealed that active MERTK, ILK and Syk were found throughout efferocytic cups, whereas AKT, FAK and SFK were concentrated along the leading edge of efferocytic cups (Fig. 3C–G; Fig. S2G–L). Consistent with the GSDM clustering data, the active signaling molecules were largely localized to discrete puncta within the efferocytic cups. Combined, these data demonstrate that MERTK is structured into preformed 180 nm diameter complexes alongside several integrins, ILK and SFK, with these molecules spatially regulated within forming efferocytic cups.

### MERTK forms an efferocytic synapse via a novel signaling pathway

**Fig. 4.**
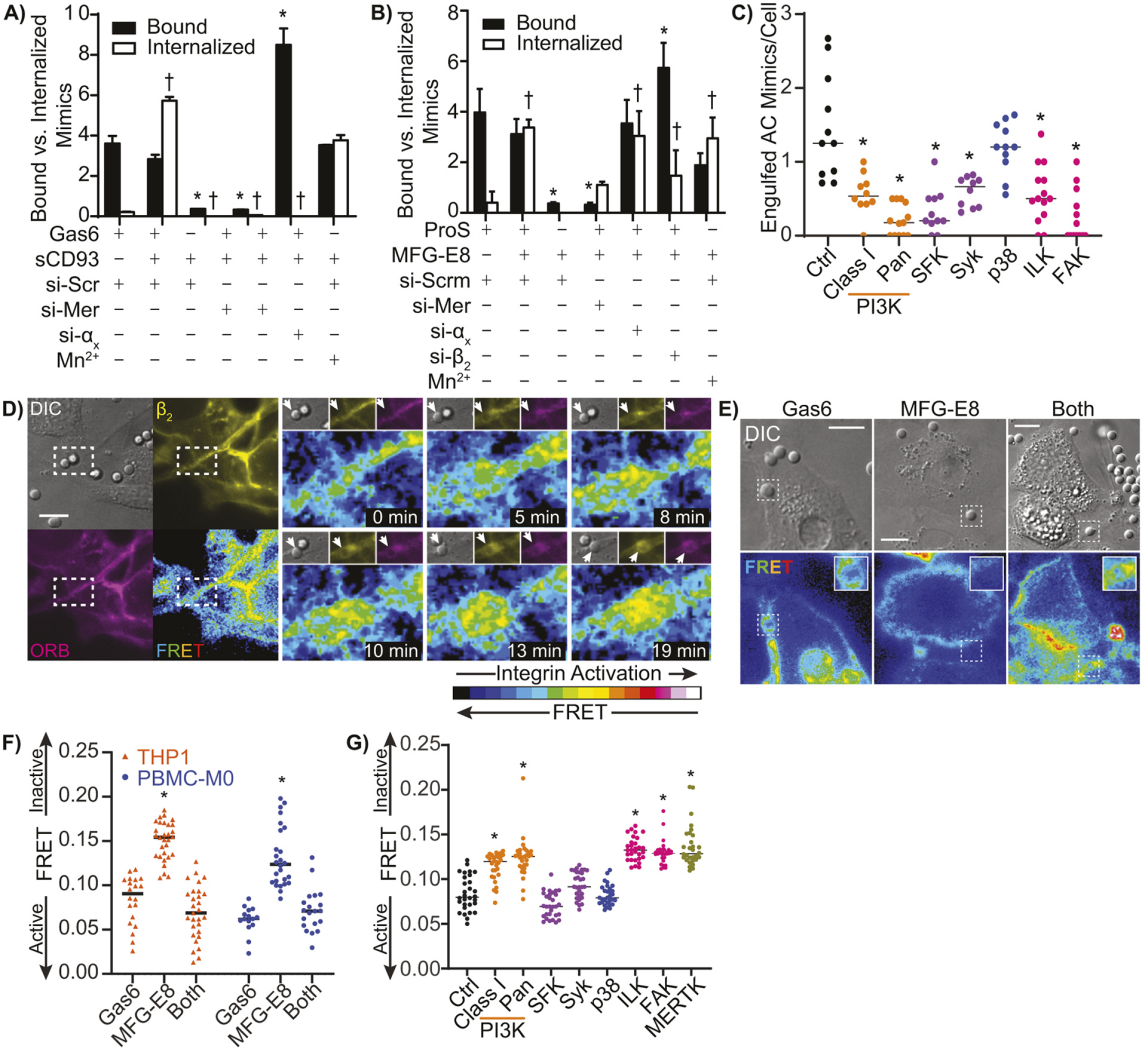
**MERTK regulates integrin affinity through a novel signaling pathway.** (A,B) Binding and internalization of apoptotic cell mimics opsonized with the opsonins (A) Gas6 and sCD93, or (B) ProS and MFG-E8, by THP1 macrophages that are either wild type (WT), treated with a scrambled (non-targeting) siRNA (si-Scr), MERTK-depleting siRNA (si-Mer), α_x_ integrin-depleting siRNA (si-α_x_), β_2_ integrin-depleting siRNA (si-β_2_), or where integrins are forcibly activated by addition of 1 mM MnCl_2_ (Mn^2+^). (C) Engulfment of apoptotic cell mimics opsonized with Gas6 and MFG-E8 by THP1 macrophages treated with inhibitors of class I PI3K (LY294002), pan-PI3K (Wortmanin), SFKs (PP1), Syk (Syk-I), p38 MAPK (SB203580), ILK (Cpd-22), FAK (PF-00562271), or a vehicle control (Ctrl). (D) Representative FRET time-lapse of β_2_ integrin activation in response to MERTK-specific (Gas6-opsonized) apoptotic cell mimics. Left, low-magnification field of the region under observation at 0 min showing white-light (DIC), β_2_ integrin (yellow), ORB (magenta), and FRET channels. Right, time-series of the region indicated by the white box, showing an increase in integrin activation beneath the apoptotic cell mimicking bead (arrows). Images representative of five repeats. Scale bar: 10 µm (the magnified views are 14.86×10.29 µm). (E,F) FRET images (E) and quantification (F) of β_2_ integrin activation on THP1-derived macrophages (orange) and PBMC-M0 macrophages (blue) in the an ROI defined by the bead in the DIC image, with the beads opsonized with Gas6, MFG-E8 or both opsonins. Scale bars: 10 µm. (G) β_2_ integrin activation in efferocytic cups forming around beads opsonized with Gas6 and MFG-E8 in THP1 macrophages treated with the same inhibitors as in C, plus an inhibitor of MERTK (UNC2250), as measured by FRET microscopy. Line in graphs show the mean; *n*=a minimum of 30 cells/condition imaged in three independent experiments. *P<0.05 compared to bound Gas6/ProS-opsonized beads bound by wild-type or vehicle-control treated macrophages (A–C,G), or to macrophages engaging a Gas6-opsonized bead (F), ^†^*P*<0.05 compared to Gas6-opsonized or ProS-opsonized beads internalized by wild-type macrophages (A,B) (Kruskal–Wallis test with Dunn's multiple comparisons test).

Given the high degree of MERTK–α_x_β_2_ integrin co-clustering, and previous reports indicating that MERTK requires integrins for its function, we next investigated the roles of MERTK and α_x_β_2_ integrin in efferocytosis by THP1-derived macrophages, using apoptotic cell mimics opsonized either with the MERTK opsonin Gas6, the α_x_β_2_ integrin opsonin sCD93 or both opsonins, with both the binding and internalization of the mimics quantified. Consistent with our observation that MERTK is the predominant efferocytic receptor on macrophages (Fig. 1), neither binding nor internalization occurred when mimics lacked the MERTK opsonin Gas6 or when MERTK was siRNA depleted (Fig. 4A). Mimics were bound by macrophages when both Gas6 and MERTK were present, but engulfment required the additional presence of both sCD93 and α_x_β_2_ integrin (Fig. 4A; Fig. S1B). This finding suggests that MERTK activates integrins upon recognition of an apoptotic cell, with the integrins then providing the necessary affinity and/or physical forces required for engulfment. Consistent with this model, mimics bearing only the α_x_β_2_ integrin ligand sCD93 were bound and internalized when integrins were forced into their high-affinity conformation by the addition of Mn^2+^ (Fig. 4A). MERTK and other TAM-family efferocytic receptors can bind to apoptotic cells via other opsonins, including ProS, which might allow the other TAM members to participate in efferocytosis. Likewise, the opsonin MFG-E8 can be ligated by multiple integrins, potentially allowing additional integrins to participate in macrophage-mediated efferocytosis. To test these possibilities, we repeated the opsonization and siRNA knockdown analyses using apoptotic cell mimics opsonized with the TAM opsonin ProS and the integrin opsonin MFG-E8 which is recognized by α_m_β_2_, α_v_β_3_ and α_v_β_5_ (Chiang et al., 2021; Fricker et al., 2012; Hirano et al., 2017). Despite ProS having a higher affinity for Axl and Tyro3 than for MERTK (Tsou et al., 2014), binding of the ProS and MFG-E8 targets was dependent on MERTK, and target engulfment was dependent on the presence of both MERTK and integrin opsonins (Fig. 4B). Unlike with sCD93, knockdown of α_x_ did not prevent engulfment, whereas β_2_ knockdown partially abrogated engulfment, consistent with multiple integrin subfamilies binding MFG-E8 (Fig. 4B).

Based on our MS and signaling pathway analyses (Table 1, Fig. 3), we identified several signaling molecules potentially important for the MERTK-mediated efferocytosis – specifically, PI3K, SFKs, Syk, p38 MAPK family proteins (downstream of MAP4K4), and the integrin-associated signaling molecules ILK and FAK. Using pharmacological inhibitors of these proteins and serum-opsonized apoptotic cell mimics, we determined that all but p38 MAPK were required for efficient engulfment by THP1-derived macrophages (Fig. 4C). This signaling pathway is reminiscent of FcγR-mediated phagocytosis, so as a comparison we analyzed the impact of these inhibitors on the phagocytic engulfment of IgG-opsonized pathogen mimics. Unlike MERTK-mediated efferocytosis, FcγR-mediated phagocytosis required only PI3K, SFKs and Syk (Fig. S3A). Thus, MERTK mediates efferocytosis via a signaling pathway that diverges from canonical phagocytic signaling. To determine whether MERTK was activating integrins, and the role of these pathways in mediating integrin activation, we probed the activation of β_2_ integrin using sensitized emission fluorescence resonance energy transfer (FRET) between a β_2_ integrin headgroup-specific FITC-labeled antibody and the membrane dye octadecyl-rhodamine B (ORB) (Lefort et al., 2009). In the low affinity (bent) conformation, the antibody will be located near the membrane, allowing for FRET to occur between FITC and the ORB in the cell membrane. Activation of the integrin induces the extended high-affinity confirmation, thus moving the headgroup away from the membrane and decreasing the FRET signal (Fig. S3B). Using live-cell FRET microscopy we analyzed the dynamics of integrin activation. Prior to contacting the Gas6-opsonized apoptotic cell mimic, THP1-derived macrophages exhibit extensive FRET throughout their plasma membrane, indicating that the β_2_ integrins are largely in an inactive conformation (Fig. 4D). As the macrophage engages the mimic, a cup-like structure forms in which there is a concurrent loss of FRET signal, indicating an activation of integrins within the cup (Fig. 4D; Fig. S4, Movie 1). As these mimics lack any integrin opsonins, this integrin activation must occur via Gas6–MERTK signaling. Consistent with MERTK being responsible for this integrin activation, both THP1-derived and PBMC-M0 macrophages bound beads opsonized with Gas6 with or without MFG-E8, followed by the activation of integrins proximal to the bound bead (Fig. 4E,F). Beads opsonized only with the integrin opsonin MFG-E8 rarely interacted with macrophages, with no integrin activation observed near these beads, indicating that MERTK ligation is a prerequisite for integrin activation (Fig. 4F). Interestingly, MERTK-mediated integrin activation within the efferocytic cup required PI3K, ILK, FAK and the kinase activity of MERTK, but not SFKs or Syk (Fig. 4G), indicating that SFKs and Syk mediate integrin-independent aspects of efferocytosis.

**Fig. 5.**
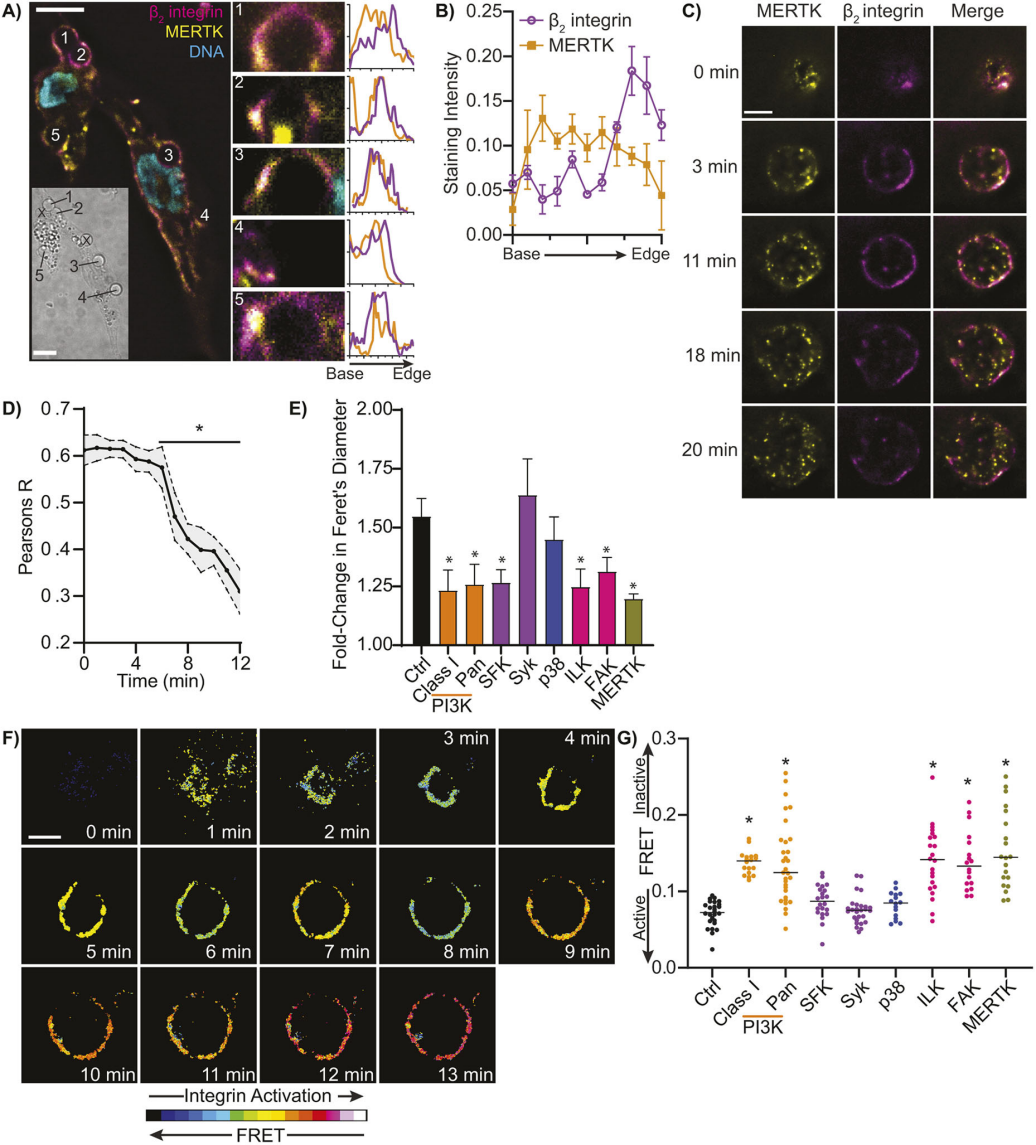
**MERTK signaling is required for efferocytic synapse formation.** (A) 3D reconstructions of efferocytic cups forming around 5 μm efferocytic targets opsonized with MFG-E8 and Gas6 in THP1-macophages labeled for MERTK (yellow), β_2_ integrin (magenta) and DNA (cyan). Left panel shows a single *z*-plane 1.5 μm above the basolateral surface of the cell with efferosomes (numbered structures) at various stages of engulfment; insert shows a DIC image of the same region with non-interacting efferocytic targets indicated by ‘X’. Middle panels show axial cross-sections of the numbered efferosomes, showing nascent efferosomes (#4), partial engulfment (#2, #3), and just following engulfment (#1, #5). Right panels show the intensity of MERTK and β_2_ integrin staining from the base to the leading edge of each efferosome. Intensity is scaled from minimum to maximum intensity, the *x*-axis of the plots are scaled 1 μm/division. (B) Quantification of MERTK (yellow) and β_2_ integrin (magenta) staining intensity from the base to leading edge of 60 efferocytic cups forming around efferocytic targets opsonized with MFG-E8 and Gas6. Intensity is expressed as fraction of integrated intensity, and distance (*y*-axis) as fractional length. (C) Timelapse micrograph showing the localization of MERTK (yellow) and β_2_ integrin (magenta) during the formation of an efferocytic synapse forming on a planar apoptotic cell mimic opsonized with MFG-E8 and Gas6 by a THP1-derived macrophage on a planar apoptotic cell mimic. (D) Quantification of MERTK colocalization with β_2_ integrin during efferocytic synapse formation on a planar apoptotic cell mimic opsonized with MFG-E8 and Gas6. (E) Quantification of the effect of PI3K, SFKs, Syk, p38 MAPK, FAK, ILK, MERTK kinase domain inhibition on the formation of an efferocytic synapse formation on planar apoptotic cell mimics opsonized with MFG-E8 and Gas6. Data are quantified in the fold-change of the maximum Feret's diameter of the synapse compared to *t*=0. (F) Activation of β_2_ integrin in an efferocytic synapse forming on an opsonized planar apoptotic mimic comprised of a Gas6 and MFG-E8 opsonized lipid planar bilayer composed of 20:80 PtdSer:PtdChol. Integrin activation is quantified by FRET microscopy. (G) Effect of inhibitors of PI3K [LY294002 (class I) and Wortmannin (Pan)], SFK (PP1), Syk (SykI), p38 MAPK (SB), ILK (ILK-I), FAK (PF), MERTK (UNC), and vehicle control on β_2_ integrin activation on opsonized planar apoptotic mimics. Data are plotted as mean±s.e.m. (B,D,E), as individual synapses with mean (G), or as representative micrographs of individual cells (A,C,F), 0 min is the time when the cell fist enters the same focal plane as the planar apoptotic cell mimic. *n*=5 (A–E) or 4 (F,G). *P<0.05 compared to 0 min (D) or untreated (E,G) (Kruskal–Wallis test with Dunn's multiple comparisons test). Scale bars: 10 μm.

We investigated the distribution of MERTK and β_2_ integrins during engulfment by generating 3D reconstructions of THP1 macrophages fixed shortly after the initiation of efferocytosis of large efferocytic targets (5 μm beads opsonized with Gas6 and MFG-E8), thus capturing targets at varying stages of engulfment (Fig. 5A). Interestingly, regardless of how far engulfment had progressed, we consistently observed an enrichment of β_2_ integrins at the leading edge of the efferocytic cup, whereas MERTK was distributed throughout the cup (Fig. 5A,B). Clustering of both MERTK and β_2_ integrins appeared to occur within the efferocytic cup, but due to the low axial resolution of fluorescent microscopy, it was not possible to image these clusters with sufficient resolution for a more detailed analysis. Thus, a ‘frustrated efferocytosis’ model – wherein THP1 macrophages are ‘parachuted’ onto planar supported lipid bilayers composed of the same lipid mixture as our apoptotic cell mimics – was used to perform a higher resolution investigation of the spatial organization of MERTK and β_2_ integrin during efferocytosis (Kovari et al., 2016; Lin et al., 2016). Macrophages form an efferocytic synapse when they contact these planar apoptotic cell mimics (Fig. 5C; Movie 2). Formation of this synapse was dependent on the presence of PtdSer and appropriate opsonins, as well as on all tested signaling molecules and on integrin activation (Fig. S5A–C). Within this synapse, MERTK and β_2_ integrin were initially colocalized, but very quickly moved into separate spatial domains (Fig. 5C,D). As was observed with 3D efferocytic targets, integrins became enriched in an expanding ring at the leading edge of the synapse, whereas MERTK was distributed throughout the synapse (Fig. 5C,D; Movies 2, 3). The expansion of the synapse was dependent on PI3K, SFKs, FAK, ILK and an active MERTK kinase domain (Fig. 5E), but interestingly did not require Syk. This is unexpected, as in canonical FcγR-mediated phagocytosis, Syk is central to the induction of the actin reorganization required to engulf pathogens (Crowley et al., 1997). We quantified β_2_ integrin activation within the synapse using FRET. At early timepoints integrins are in their inactive state and were rapidly activated as the synapse formed (Fig. 5F; Movie 4). This integrin activation required the same signaling molecules as did integrin activation in response to bead-based mimics (Fig. 5G). In RPE cells, MFG-E8 binding induces MERTK cleavage at certain points in the circadian cycle (Law et al., 2015), but we were unable to detect soluble MERTK in our macrophage supernatants, indicating that this is either an RPE cell-specific phenomena or that minimal cleavage occurred over the observation period (data not shown). Although these data show that MERTK signaling activates integrins and coordinates synapse formation, the mechanisms which underly the formation of the synapse and the spatial separation of MERTK and integrins are unknown.

### Diffusion shapes the efferocytic synapse

**Fig. 6.**
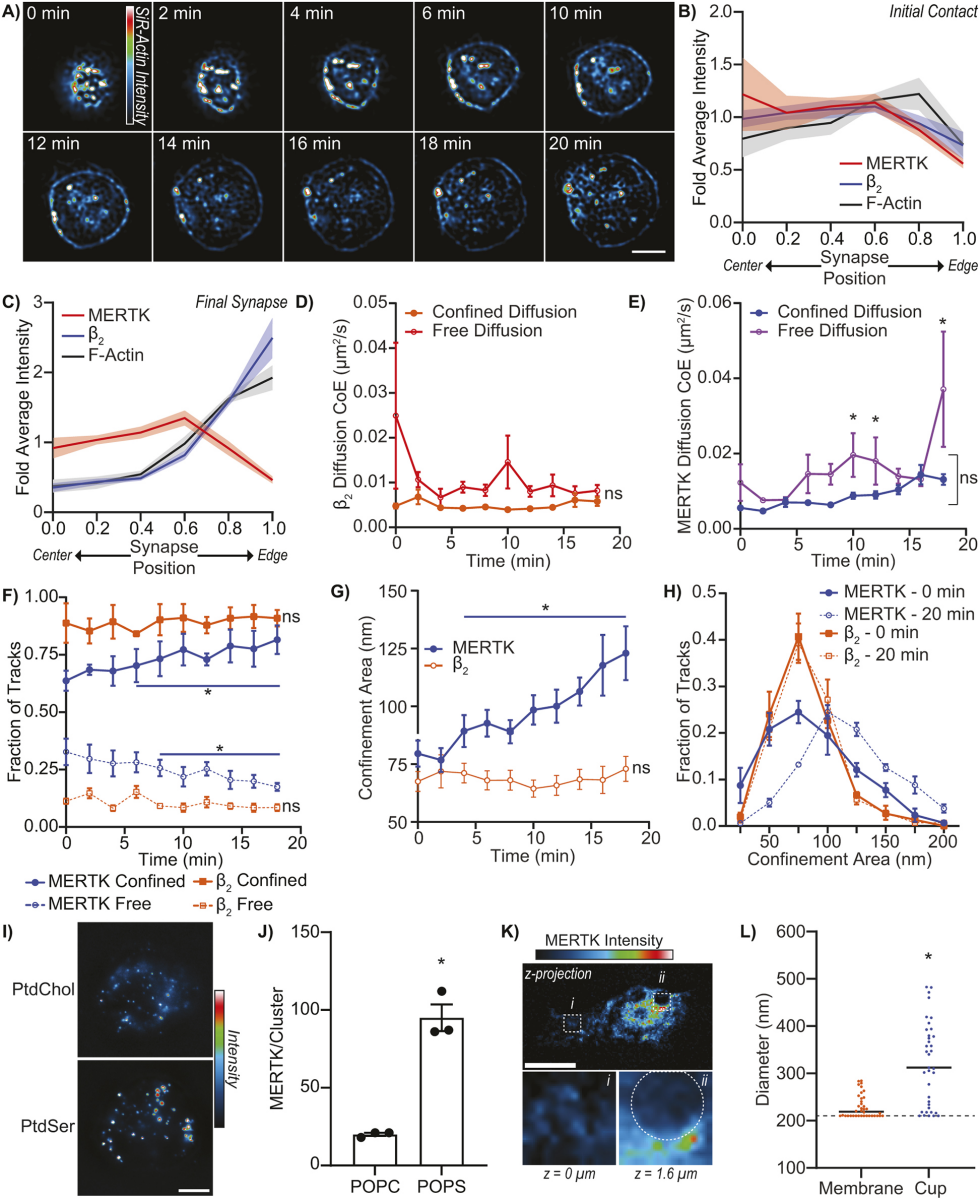
**Changes in diffusion structures the efferocytic synapse.** Efferocytic synapse formation was quantified on apoptotic cell-mimicking planar lipid bilayers comprising 20% PtdSer and 80% PtdChol (PtdSer) opsonized with MFG-E8 and Gas6 or on bilayers mimicking a healthy cell (100% PtdChol). (A) SRRF image of actin distribution, labeled with SiR–Actin, during efferocytic synapse formation on a PtdSer-containing bilayer. (B,C) Radial distribution of MERTK, β_2_ integrin and F-actin in macrophages immediately after contacting a PtdSer-containing bilayer (B) and once efferocytic synapse formation was complete (C, 12 min post-contact). (D,E) Changes in the diffusion rate of β_2_ integrins (D) and MERTK (E) during efferocytic synapse formation on a PtdSer-containing bilayer. Diffusion has been classified as either free diffusion or confined diffusion coefficient (CoE) using moment scaling spectrum analysis. (F) Quantification of the portion of MERTK and β_2_ integrin undergoing free diffusion (free) versus confined diffusion during efferocytic synapse formation on a PtdSer-containing bilayer. (G) Changes in the size of the confinement areas restricting the diffusion of MERTK and β_2_ integrin during efferocytic synapse formation on a PtdSer-containing bilayer. (H) Size distribution of MERTK and β_2_ integrin confinement zones before (0 min) and after (20 min) efferocytic synapse formation on a PtdSer-containing bilayer. (I,J) Images (I), and stepwise photobleaching analysis of the number of MERTK per cluster (J) on THP1-derived macrophages 20 min after contacting PtdChol versus PtdSer-containing bilayers. (K) MERTK cluster size on PBMC-M0 cells within the bulk cell membrane versus within an efferocytic cup. Top panel is a maximum-intensity *z*-projection of all MERTK on the macrophage, inset *i* is of the basolateral membrane of the cell at a location distal to the efferocytic cup, inset *ii* is located at the base of the forming efferocytic cup with the position of the apoptotic cell mimic indicated by the dotted circle. (L) Quantification of MERTK cluster size within the bulk membrane of PBMC-M0 cells versus within the base of efferocytic cups forming around 3.5 μm diameter efferocytic targes. Dashed line indicates the radial resolution of the microscope used for quantification. Data are presented as mean±95% confidence interval (B–H), mean±s.e.m. (J), and median (L). *n*=minimum of 5. 0 min is when the cell fist enters the focal plane. *P<0.05; ns, not significant compared to *t*=0 [one-way ANOVA with Tukey correction (E–G) or Mann–Whitney *U*-test (J,L)]. Scale bars: 10 μm.

There must be a difference in the movement of MERTK and β_2_ integrin within the synapse to drive their separation into separate spatial domains. Proteins in the plasma membrane diffuse via two distinct diffusive patterns – freely diffusing proteins that approximates Brownian diffusion, and proteins whose diffusion is confined to small sub-regions of the plasma membrane (Jaqaman et al., 2011). These confinement zones – termed ‘corrals’ – are created by ‘picket-fences’ consisting of cortical actin ‘fences’ and actin-anchored transmembrane protein ‘pickets’ (Kusumi et al., 2005). Given that active integrins bind to actin via talins (Klapholz and Brown, 2017), changes in the actin cytoskeleton might serve to both form the synapse and to separate MERTK from integrins. As corrals are often smaller than the ∼250 nm resolution of light microscopy, we imaged the distribution of actin in THP1 macrophages with 95 nm resolution using SRRF microscopy. Actin was found in a relatively even meshwork beneath the plasma membrane before synapse formation, but following contact with the bilayer this actin was readily redistributed to a ring at the edge of the synapse (Fig. 6A–C; Movie 5). This change in actin distribution was dependent on the presence of PtdSer and was not observed on PtdChol bilayers (Fig. S5D–G; Movie 6), and synapse formation was dependent on active endocytosis and exocytosis pathways (Fig. S5H). As expected, MERTK remained distributed throughout the synapse during synapse formation, whereas β_2_ integrin localized to the leading edge (Fig. 6B,C).

Given the distribution of actin in the synapse, and the role of actin in confining the diffusion of transmembrane proteins, we expected to observe confined diffusion of β_2_ integrin at the synapse edge and highly diffusive MERTK in the synapse center (Freeman et al., 2018; Kusumi et al., 2005). Consistent with this model, diffusion analysis by single-particle tracking microscopy revealed that β_2_ integrins had low diffusivity and remained in small corrals throughout synapse formation, whereas the diffusivity of MERTK increased as synapse formation progressed (Fig. 6D–G). This pattern of high β_2_ integrin confinement and small confinement zone size is consistent with the integrins remaining within actin-dense regions of the synapse, possibly due to direct anchoring to actin by talins and FAK (Schwartz, 2011). Paradoxically, despite being localized to an area of the synapse which undergoes actin depletion during synapse formation, and exhibiting the increase in diffusivity expected in areas of low actin density, the portion of MERTK undergoing confined diffusion increased significantly as synapse formation progressed (Fig. 6F). Even more paradoxically, this increase in confined diffusion was accompanied by a near-doubling of the size of the MERTK confinement zones (Fig. 6G). This increase in confinement region size occurred monotonically across all MERTK molecules in the synapse, indicating a change in the mechanism that confines MERTK diffusion rather than the appearance of a second MERTK population confined by a different mechanism (Fig. 6H). The ∼15% increase in the portion of confined MERTK and the doubling of the confinement zone size following synapse formation represents a dramatic change in the diffusive properties of MERTK, far larger than similar changes reported for other receptors following ligand binding (Lopes et al., 2017; Sugiyama et al., 2023; Wong et al., 2014). On substrates lacking PtdSer, β_2_ integrin and MERTK exhibited no change in their diffusivity, in the fraction of molecules undergoing free versus confined diffusion or in confinement zone size (Fig. S5E–G), confirming that the changes in diffusion we observed on PtdSer-containing substrates was due to efferocytic signaling and not due to contact-dependent cellular responses or crowding effects following cell–substrate contact.

Given the increase in MERTK confinement zone size, and the increase in MERTK diffusivity within these confinement zones, it seemed likely that the increase in MERTK diffusional confinement was due either to ligand-mediated immobilization of MERTK or due to clustering-induced immobilization. The former is unlikely given that apical-face lipids in supported lipid bilayers are highly diffusive and experience little diffusional confinement (Rose et al., 2015), whereas the latter is frequently observed (Marchetti et al., 2013; Su et al., 2021). To test whether MERTK was forming larger clusters within the synapse, we used stepwise photobleaching to measure the relative number of MERTK present in MERTK clusters on cells contacting PtdSer-containing versus PtdSer-free planar bilayers (Fig. 6I). Quantification of MERTK cluster size by stepwise photobleaching analysis confirmed that MERTK was undergoing ligand-induced receptor clustering, with MERTK clusters increasing over four times in size on PtdSer-containing planar bilayers (Fig. 6J). To confirm this increase in clustering was not a cell line artifact, this analysis was repeated on PBMC-M0 cells, which displayed a similar increase in MERTK clustering during efferocytosis, with MERTK clusters expanding from below the lateral resolution limit of our microscope to clusters ∼320 nm in diameter (Fig. 6K,L). Combined, these data support a model wherein β_2_ integrins define the leading edge of the forming efferocytic cup and are retained on the leading edge via either trapping in the dense cortical actin cytoskeleton or via direct linkage to actin. In contrast, MERTK is retained in the center of the cup by ligand-induced clustering which results in the immobilization of the MERTK–ligand clusters.

## DISCUSSION

Although nearly a dozen efferocytic receptors are expressed by macrophages, MERTK is the predominant efferocytic receptor used by these cells, and is the primary or sole efferocytic receptor in multiple tissues including the heart and eyes (Cai et al., 2017; Feng et al., 2002; Seitz et al., 2007; Thorp et al., 2008). SNPs in MERTK and its opsonins predispose individuals to a range of inflammatory and autoimmune disorders, highlighting the importance of this receptor to human health (Binder et al., 2016; Hurtado et al., 2010; Ma et al., 2011). In this study, we have demonstrated that MERTK activates a signaling pathway via its intrinsic kinase domain that is dependent on PI3K, SFK, ILK and FAK. The resulting PI3K activity induces the inside-out activation of β_2_ integrins, which then bind to apoptotic cells and mediate the formation of an efferocytic cup via an SFK-, ILK- and FAK-dependent pathway. The differential diffusion of MERTK and β_2_ integrins is required for proper structuring of this structure, with the diffusional confinement of β_2_ integrins retaining them at the actin-rich edge of the cup, and ligand-induced clustering leading to retention of MERTK in the actin-poor center.

The efferocytic synapse formed by MERTK is reminiscent of the phagocytic synapse formed during antibody-mediated phagocytosis by FcγRs. This phagocytic synapse forms a similar actin ring, which in early phagocytosis serves to expand the synapse, and later in phagocytosis serves as a ‘jaw’ that exerts constrictive forces on the phagocytic target that drive phagosome closure (Barger et al., 2022; Francis et al., 2022; Jaumouillé et al., 2019; Vorselen et al., 2021). The efferocytic and phagocytic synapses also contain a similar bounding ring of integrins, which during IgG-mediated phagocytosis serves to exclude proteins with a large glycocalyx from the phagocytic synapse (Freeman et al., 2016). This process excludes inhibitory phosphatases, such as CD45 and CD148, in a manner similar to the exclusion of phosphatases from the T cell synapse (Cordoba et al., 2013). Although the efferocytic synapse shares a similar bounding ring of integrins, it is unlikely that these integrins are playing the same role. Indeed, at the time of this writing there are no published reports showing a role for CD45 or CD148 in the inhibition of efferocytosis. Moreover, phagocytosis needs to be sensitive to the ligation of a small number of receptors in order to ensure the removal of poorly opsonized or small pathogens, with the exclusion of phosphatases from small binding regions allowing for unopposed activation of phagocytic signaling (Freeman et al., 2016). In contrast, efferocytosis needs to be tightly regulated, as the ‘eat me’ signal PtdSer is frequently exposed on non-apoptotic cells, including on platelets during coagulation, on degranulating mast cells, on migratory CD45RB^lo^ T cells, on CD8^+^ T cells following antigen recognition, in skeletal muscle, on activated and IL-10 producing B cells, and following galectin binding to neutrophils (Audo et al., 2017; Dillon et al., 2000; Elliott et al., 2005; Fischer et al., 2006; Lentz, 2003; Smrž et al., 2007; Stowell et al., 2007; Thiagarajan and Tait, 1990; van den Eijnde et al., 2001). This regulation is mediated by the ‘don't eat me’ receptor CD47 on non-apoptotic cells and its cognate receptor SIRPα on the efferocyte (Vernon-Wilson et al., 2000). Unlike CD45 and CD148, neither CD47 nor SIRPα are excluded from the efferocytic synapse, thus ensuring that their inhibitory functions are retained even when efferocytic ligands are present (Morrissey et al., 2020). Instead of relying on exclusion, this inhibitory signaling is regulated by CD47 clustering, with efferocytosis occurring after the apoptosis-induced dispersion of CD47 clusters (Lv et al., 2015).

Although integrins are not acting as an exclusionary barrier for CD47 or SIRPα in the efferocytic synapse, they do serve a crucial role in efferocytosis, as our data indicate that MERTK is unable to mediate the engulfment of apoptotic cells in the absence of integrin ligands, or when MERTK-mediated integrin activation is inhibited (Morrissey et al., 2020). Although conjectural, we believe that MERTK is utilizing integrins to mediate high-affinity binding and actin reorganization during efferocytosis. Indeed, the equilibrium dissociation constant of integrin–ligand pairs is typically in the range of 10^−9^ to 10^−10^ M, which is two orders of magnitude higher than the affinity of MERTK and other TAMs for Gas6 and Protein S, with integrin clustering further increasing the avidity of this interaction (Chigaev et al., 2001; Connors et al., 2007; Hirano et al., 2017; Li et al., 2021). Moreover, integrins can independently mediate the actin reorganization processes required for engulfment of large particulate targets, most notably in the form of complement-mediated phagocytosis where the entirety of the phagocytic process is mediated by β_2_ integrin–iC3b interactions and integrin signaling (reviewed in Freeman and Grinstein, 2014). Further evidence that MERTK is utilizing the integrin-mediated phagocytosis pathway can be found in the signaling molecules required for MERTK-mediated efferocytosis. Herein, we determined that the kinase Syk was not required for MERTK-mediated efferocytosis, whereas ILK and FAK – which are canonically associated with integrin signaling – were essential. Complement-mediated phagocytosis displays the same independence from Syk signaling, whereas Syk is required for antibody-mediated phagocytosis (Kiefer et al., 1998; Walbaum et al., 2021). Likewise, FAK signaling is inhibitory for antibody mediated phagocytosis, whereas both FAK and ILK are required for integrin-mediated phagocytosis (Antonieta Cote-Vélez et al., 2001; Sayedyahossein et al., 2012; Shiratsuchi and Basson, 2004). Crucially, we were able to induce the engulfment of sCD93-opsonized (e.g. α_x_β_2_ integrin-specific) efferocytic targets simply by forcing integrin activation by the addition of Mn^2+^, and we were able to abrogate integrin activation in the presence of opsonized efferocytic targets through inhibiting MERTK kinase activity or by knocking down MERTK. Consistent with efferocytic receptors activating integrins in order to induce engulfment, ligated SIRPα inhibits efferocytosis by preventing integrin activation (Morrissey et al., 2020). Combined, these data indicate that MERTK acts as a sentinel receptor that identifies apoptotic cells to then engage the canonical integrin-mediated phagocytosis pathway to drive engulfment.

Previous studies analyzing other efferocytic receptors have found a similar dependence on integrins, leading to the tether model, which proposed that efferocytic receptors such as MERTK act as passive ‘tethers’ that stabilize the association of apoptotic cells with efferocytes, thereby providing integrins the time necessary to recognize and bind to the apoptotic cell (Nishi et al., 2014; Toda et al., 2012). Our data challenges this model, and indeed, demonstrates that MERTK generates a signal via its kinase domain which activates PI3K, which in turn activates integrins and allows efferocytosis to proceed. Without the activating signals from the MERTK kinase domain and PI3K, neither integrin activation nor engulfment of apoptotic targets occurs. Although this is not the first indication that efferocytic receptors are more than passive receptors (Flannagan et al., 2014), to our knowledge our data is the first identifying a specific signaling pathway induced by an efferocytic receptor that then regulates integrin affinity and activation. Interestingly, multiple other efferocytic receptors are known or suspected to associate with MERTK or another member of the TAM family, for example, TIM4 and BAI1 with MERTK, and TIM1 with Tyro3 (Brouillette et al., 2018; Moon et al., 2020; Penberthy et al., 2017), with these interactions often having a synergistic effect on efferocytosis. Neither the TIM family of receptors nor BAI1 have intrinsic kinase domains, suggesting that they might act as classical tethers and/or require the kinase activity of TAM family kinases to initiate signaling. Indeed, although BAI1 was found to interact with the ELMO–DOCK180 complex that initiates the actin reorganization required for phagocytosis and efferocytosis, the activity of this complex is initiated by a tyrosine-kinase-mediated phosphorylation of CrkII, with TAMs known to be capable of providing this activating phosphorylation (Lee et al., 2007; Park et al., 2007; Wu et al., 2005). Although our MS analysis did not identify any proteins known to act as intermediaries between MERTK and PI3K, MERTK is known to directly phosphorylate and activate the PI3K effector AKT, and immunoprecipitations using a soluble MERTK kinase domain as bait identified the scaffolding protein Grb2 as a major interactor (Jiang et al., 2019; Shelby et al., 2013). Grb2 is known to act as a bridge between phosphorylated receptor tyrosine kinases and PI3K, and indeed, Grb2 has been shown to bind to the active MERTK kinase domain where it mediates recruitment and activation of type 1A PI3Ks through interactions with the SH2 domain in the p85 subunit of PI3K (Georgescu et al., 1999). Although most studies identify PI3K signaling as a product of outside-in signaling through integrins, our observation of PI3K-mediated inside-out activation is not unprecedented. Thamilselvan et al. identified a pressure-induced pathway resulting in the PI3K-dependent activation of β_1_ integrins, whereas both CD44-mediated phagocytosis and LFA-1-mediated T cell adhesion occur via the PI3K-dependent activation of the GTPase Rap-1, which then activates β_2_ integrins (Garçon and Okkenhaug, 2016; Thamilselvan et al., 2007; Vachon et al., 2007).

In summary, we have identified the sequence of events by which MERTK activates integrins and mediates the formation of a functional efferocytic synapse. MERTK ligation induces activation of its kinase domain, which activates PI3K via Grb2 (Shelby et al., 2013). PI3K activity then induces the inside-out activation of integrins that then mediate the actin reorganization needed for expansion of the synapse around the apoptotic cell in a SFK-, ILK- and FAK-dependent manner. Given the structural similarities of MERTK with the other TAM family members Axl and Tyro3, this mechanism is likely universal across this family of efferocytic receptors.

## MATERIALS AND METHODS

### Materials

THP1 cells were obtained from Cedar Lane Labs (Mississauga, Canada). Roswell Park Memorial Institute (RPMI), Dulbecco's modified Eagle's medium (DMEM) and fetal bovine serum (FBS) were purchased from Wisent (Saint-Jean-Baptiste, Canada), trypsin-EDTA and antibiotic-antimycotic were purchased from Corning (Manassas, Virginia, USA). All antibodies used in this study are described in Table S1. Round coverslips (#1.5 thickness 18 mm diameter) and 16% paraformaldehyde (PFA) were purchased from Electron Microscopy Supplies (Hatfield, Pennsylvania, USA). All tissue culture plates, centrifuge tubes, plastic ware, wheat germ agglutinin-Alexa Fluor 647, accutase, octadecyl rhodamine B, Hoechst 33342, Permafluor mounting medium, N-hydroxysuccinimidobiotin and cell proliferation dye 670 were purchased from Thermo Fisher Scientific. Silica beads were purchased from Bangs Laboratories (Fishers, Indiana, USA). Lipids, biotin PE and the liposome extruder were from Avanti Polar Lipids (Alabaster, Alabama, USA). M-CSF and Gas6 was purchased from R&D (Minneapolis, MN, USA). ILK inhibitor Cpd-22 was purchased from EMD Millipore. The soluble MERTK ELISA kit was from Abcam. The FAK inhibitor PF-00562271 was purchased from Sigma-Aldrich, and the other pharmacological inhibitors for Src-family kinases (PP1), Syk (Syk Inhibitor I), class-I PI3K (Ly294002), pan-PI3K (Wortmannin) and p38 MAPK (SB 203580) were purchased from Cayman Chemical (Ann Arbor, Michigan, USA). Phorbol 12-myristate 13-acetate (PMA) and rest of the chemicals were purchased from BioShop Canada (Mississauga, Canada). ON-TARGET siRNAs against MERTK, Tyro3, Axl and α_x_ integrin were purchased from Horizon Discovery (Cambridge, UK). Soluble CD93 was produced as described previously (Blackburn et al., 2019). 4–20% precast SDS-PAGE gels, Laemmli buffer and colloidal Coomassie stain was from Bio-Rad (Hercules, California). SiR-actin was from Spirochrome (Stein am Rhein, Switzerland). Accell cell-penetrant siRNA and RNA medium were from Horizon Biosciences (UK). Partek Flow was from Parteck (St. Louis, MO, USA) MATLAB and Prism software were purchased from MathWorks (Natick, Massachusetts, USA) and GraphPad (La Jolla, California, USA), respectively. FIJI was downloaded from https://fiji.sc/ (Schindelin et al., 2012).

### THP1 cell culture and differentiation

THP1 cells were maintained in RPMI supplemented with 10% FBS at 37°C in 5% CO_2_. Cells were split upon reaching 1.5×10^6^ cells/ml. To differentiate THP1 cells into macrophages, cells were collected by centrifugation (250 ***g*** for 5 min) and resuspended in fresh medium at 2.5×10^5^ cells/ml and 100 ng/ml PMA added. These cells were then seeded into 12-well tissue culture plate with #1.5 thickness 18 mm diameter coverslips plated into each well at a density of 2.5×10^5^ cells/well and differentiated for 72 h at 37°C in 5% CO_2_. PMA was removed via a medium change at least 1 h prior to all experiments to eliminate any PMA-mediated integrin activation. Cells were tested every 3 months for contamination using a HEKBlue assay.

### Human peripheral blood macrophage differentiation

The collection of blood from healthy donors was approved by the Health Science Research Ethics Board of the University of Western Ontario and was performed in accordance with the guidelines of the Tri-Council policy statement on human research. Blood was drawn into heparinized vacuum collection tubes, layered on an equal volume of Lympholyte-poly and centrifuged at 300 ***g*** for 35 min at 20°C. The top band of peripheral blood mononuclear cells was collected, washed once (300 ***g***, 6 min, 20°C) with PBS, resuspended in completed medium (RPMI-1640 plus 10% FBS and 1% antibiotic-antimycotic solution), and ∼10^6^ cells placed into each well of a 12-well plate. The cells were allowed to adhere for 1 h at 37°C and 5% CO_2_, then washed gently with PBS to remove non-adherent cells, and the cells cultured in complete medium with 10 ng/ml M-CSF (10 ng/ml) for 7 days. For siRNA treatment, for each well in a 12-well plate, 1 nmol of scrambled or MERTK-targeting cell-penetrant Accell siRNAs was diluted into 7.5 μl of siRNA buffer, and then diluted 1:10 with Accell delivery medium for a final volume of 750 μl. On the fifth day of differentiation, the medium was removed from the macrophages and replaced with the diluted siRNA and cultured for an additional 48 h. Knockdown was confirmed by RT-PCR.

### RNA-seq

Expression of efferocytic receptors in THP1 macrophages was determined by searching our previously published RNA-seq dataset for known efferocytic receptors and opsonins (Yin et al., 2020). Briefly, total RNA was isolated from PMA-differentiated THP1 macrophages and processed with a Vazyme VAHTS Total RNA-seq Library Prep Kit for Illumina, single-end reads sequenced on an Illumina NextSeq 500, 1 x76 bp, using 75 cycles of a High Output v2 kit. Fastq data files were analyzed using Partek Flow (St. Louis, MO) and the sequences were aligned to the *Homo sapiens* genome using STAR 2.6.1d and annotated using Ensembl v 98. Gene expression levels were normalized Reads Per Kilobase of transcript, per Million mapped reads (RPKM), and genes with fewer than 10 reads removed from the dataset. This dataset is available at doi:10.5683/SP2/ISOA8W.

### Mass spectrometry

As an immunoprecipitation-suitable anti-MERTK antibody was not available, for the MS experiments only, HA–MERTK-expressing cells which express HA–MERTK at levels similar to the endogenous protein were prepared as previously described (Evans et al., 2017) and cultured in DMEM plus 10% FBS at 37°C and 5% CO_2_. These cells were washed twice with PBS and then reversibly crosslinked using the ReCLIP [0.5 mM dithiobis(succinimidyl propionate) and 1.0 mM dithio-bismaleimidoethane] for 20 min at room temperature (Smith et al., 2011). Crosslinking was stopped by the addition of 1 ml of quenching buffer (5 mM L-cysteine plus 20 mM Tris-HCl, pH 7.4) for 10 min, then the cells lysed in 2 ml of RIPA buffer supplemented with 1 mM NaVO_4_, 0.25 mM PMSF, 200 nM okadaic acid, 10 mM NaF and HALT protease inhibitor cocktail. 1 µg of anti-HA was added to half the cell lysate, 1 µg of an isotype control added to the other half, and both incubated for 2 h at 4°C, at which point 20 µl of Protein A/G beads were added to each tube, and the lysates incubated for an additional hour at 4°C. The beads were pelleted by a 1 min 16,000 ***g*** centrifugation and washed three times with RIPA buffer using 1 min 16,000 ***g*** centrifugation. After the final wash, the beads were pelleted, resuspended in 50 µl of Laemmli buffer plus 350 mM DTT, and heated at 98°C for 5 min. The beads were centrifuged at 21,000 ***g*** for 5 min and the supernatant was then loaded into a 4–20% gradient SDS-PAGE gel, run at 100 V. The gel was fixed with 40% ethanol and 10% acetic acid for 15 min and then stained with colloidal Coomassie stain as per the manufacturer's instructions. Bands unique to the anti-HA lane were excised with an Ettan Robotic Spot-Picker, destained with 50% acetonitrile+50 mM ammonium bicarbonate, treated with 10 mM DTT and 55 mM iodoacetamide and digested with trypsin. Peptides were extracted with 1% formic acid plus 2% acetonitrile, dried, and then suspended in water with 0.1% trifluoroacetic acid and 10% acetonitrile. Mass spectrometry was performed on an AB Sciex 5800 TOF/TOF System and analyzed using a MASCOT database search. To limit spurious detections, only proteins present in three independent experiments were included in downstream analyses.

### Immunostaining

Unless otherwise noted, cells were first washed with PBS and fixed with 4% PFA in PBS at 10°C for 20 min. For intracellular labeling, cells were permeabilized with PBS plus 1% BSA and 0.1% Triton X-100 for 1 h at room temperature. Cells were then labeled by a 20 min (extracellular) or 1 h (intracellular) incubation with primary antibodies (1:200, anti-human MERTK; 1:40, FITC anti-human CD18; 1:100, anti-human CD11c and anti-human CD11b monoclonal antibodies; see Table S1) in PBS+0.5% BSA. Cells were then washed three times for 15 min each time with PBS and stained using fluorescently tagged secondary Fab fragments at a 1:1000 dilution in PBS plus 0.5% BSA for 20 min (extracellular) or 1 h (intracellular). Cells were washed three times for 15 min each time with PBS, and if required, incubated for 5 min with 1:10,000 Hoechst 33342 in PBS for 5 min, and washed a final time in PBS before being mounted on glass slides with PermaFluor.

### Conventional and SRRF microscopy

Unless otherwise noted, microscopy was performed on a Leica DMI6000B microscope equipped with a photometrics Evolve-512 delta EM-CCD camera, Chroma Sedat Quad filter set, heated and CO_2_ perfused live-cell stage, 100×/1.40 NA and 60×/1.40 NA objectives, adaptive focus control and running Inscoper control software. All live-cell imaging is performed in imaging buffer (150 mM NaCl, 5 mM KCl, 1 mM MgCl_2_, 100 µM EGTA, 2 mM CaCl_2_, 20 mM HEPES and 2 g/l sodium bicarbonate, pH 7.4), using the framerates indicated in each figure. Super resolution radial fluctuation microscopy (SRRF) was used for imaging actin structures below the resolution limit of conventional microscopy (<100 nm in diameter), using cells stained with SiR–actin live-cell dye as per the manufacturer's protocol. At each time point separate high-speed timelapses (50 frames, <50 ms/frame) were captured of each channel. The resulting images were imported into FIJI and SRRF images generated using the eSRRF algorithm with a magnification of 5, radius of 7, and sensitivity of 1 (Laine et al., 2022). The resulting images had a spatial resolution of 93-97 nm. The images for the individual time points were then assembled into a time-lapse video and the radial density of actin quantified in FIJI using the Radial Profile Extended plugin (Schindelin et al., 2012).

### Ground state depletion microscopy

THP1 macrophages were grown on coverslips as described above, and then fixed for 15 min at 37°C in PEM buffer (80 mM PIPES, 5 mM EGTA, 2 mM MgCl_2_, pH ∼6.9) plus 4% PFA to limit artificial receptor clustering (Pereira et al., 2019). For extracellular staining, samples were then washed once with PBS and blocked for 1 h at room temperature with PBS plus 2.5% BSA, whereas for intracellular staining cells were washed once with PBS and blocked and permeabilized for 1 h with PBS plus 2.5% BSA plus 0.1% Triton X-100. Next, samples were stained at saturating concentrations with anti-MERTK plus one or two of (depending on species and fluorophore compatibility): anti-β_2_ integrin, anti-α_x_ integrin, anti-Syk or anti-FAK and incubated for 20 min (extracellular labeling) or 1 h (intracellular labeling) followed by three 15 min washes in PBS. Samples were then stained with fluorescently labeled Fab fragments, diluted 1:1000 in PBS plus 2.5% BSA, to avoid cross-linking (Caetano et al., 2015; Caetano Crowley et al., 2019), using the same incubation times and washes as used for primary antibodies. The cells were post-fixed with 2% PFA in PEM buffer at room temperature for 20 min. Samples were immediately transferred to a chamber slide containing PBS plus 100 mg/ml β-mercaptoethylamine and transferred to the stage of a Leica GSDM microscope. GSDM acquisitions of the basolateral cell surface were acquired in TIRF mode. The resulting molecular coordinate lists were imported into our MIISR analysis software, which was then used to perform spatial association analysis, to measure inter-protein interactions, and radial distribution analyses, to quantify co-clustering. To confirm that these associations were not spurious, Monte Carlo simulations were used to mimic non-interacting proteins by randomizing protein positions over the same image area, and these analyses repeated (Caetano et al., 2015; Earl and Deem, 2008; Metropolis and Ulam, 1949). Radial distribution analysis [G(r)] values scale with protein density, thus all G(r) plots are scaled minimum–maximum.

### Preparation of phagocytic and efferocytic targets

Mimics of antibody-opsonized pathogens were prepared by mixing 10 μl of 3 μm polystyrene-divinylbenzene beads with 100 μl of PBS and 10 μl human IgG, then rotating for 2 h at room temperature. Beads were washed twice with 1 ml of PBS and a 1 min 4500 ***g*** centrifugation, then re-suspended with 100 μl of PBS. Efferocytic targets were prepared by combining 10 μl of 3.17 or 5 μm silica beads with 25 nmol of a 79.9:20:0.1 molar mixture of phosphatidylcholine (PtdChol):PtdSer:biotin-phosphatidylethanolamine (biotin-PE) in chloroform. Chloroform was then evaporated under nitrogen gas, the beads washed twice in 500 μl PBS by centrifuging at 4500 ***g*** for 1 min, and then resuspended in 100 μl PBS. Opsonized PtdSer and PtdChol beads were prepared by rotating 3 μl of the lipid-coated bead preparation with 3 μl of recombinant Gas6 and/or 5 μl of recombinant MFG-E8 in a total volume of 25 µl PBS for 2 h at room temperature and washed once with PBS. For sCD93-opsonized beads, 3 μm polystyrene/divinylbenzene beads were coated with 3 μl of recombinant Gas6 and/or 5 μl of recombinant sCD93 as above, and then any remaining protein binding sites blocked by addition of an equal volume of 1% BSA in PBS for 30 min.

### Apoptotic cell targets

Apoptotic cells were prepared as described previously (Taruc et al., 2018). Briefly, Jurkat T cells were grown in RPMI plus 10% FBS, pelleted with a 5 min 500 ***g*** centrifugation, suspended in serum-free medium containing 1 µM staurosporine at 2×10^6^ cells/ml, and cultured overnight (∼16 h) at 37°C in 5% CO_2_. The cells were then pelleted (500 ***g*** for 5 min), resuspended in 1 ml PBS plus ∼0.005 mg N-hydroxysuccinimidobiotin and 5 µM cell proliferation dye 670, and incubated at room temperature for 20 min. 10% (v/v) human serum or an equal volume of 2.5% BSA in PBS was added to quench any unused dye. If BSA was used for quenching, the apoptotic cells were subsequently opsonized by addition of 3 μl of recombinant Gas6 and/or 5 μl of recombinant sCD93 for 2 h at room temperature, and washed once with PBS. Apoptotic cells were used immediately after preparation.

### Inhibitor and siRNA treatment

For assays using pharmacological inhibitors, cells were pre-treated with 5 µM of the MERTK kinase domain inhibitor UNC2250 (Zhang et al., 2013), 2.5 μM of the class I PI3K inhibitor LY294002, 100 nM of the pan-PI3IK inhibitor Wortmannin, 30 μM of the SFK inhibitor PP1, 100 nM Syk inhibitor I, 10 µM of the p38 MAPK inhibitor SB 203580, 2 µM of the ILK inhibitor Cpd-22 inhibitor, or 25 µM of the FAK inhibitor PF-00562271 for 15 min at 37°C prior to starting the experiment, and the inhibitors were maintained at these concentrations throughout the experiment (Heit et al., 2002, 2008). For assays using siRNA, THP1 macrophages were suspended by scraping, collected by a 5 min 600 ***g*** centrifugation, and resuspended at 2×10^7^/ml in buffer R, to which 150 μM of siRNA was added and the sample electroporated using a Neon electroporation system with 2×20 ms pulses at 1400 V. The cells were then placed onto glass coverslips placed in a 12-well plate, along with 1 ml of RPMI plus 10% FBS, and allowed to recover for a minimum of 24 h in a 37°C/5% CO_2_ incubator. Knockdown was confirmed by semi-quantitative RT-PCR. Briefly, total RNA was purified and reverse transcribed, amplified using multiplex PCR primers for MERTK (5′-CCAGGTGACCTCTGTCGAATCAAA-3′, 5′-GGGATTTTTCAGGCTGTTCGTTAACA-3′), Axl (5′-CTCCACCTGGTCTCCCG-3′, 5′-CCAGGGGCACTCCCT-3′), Tyro3 (5′-CTCTTCTCATGACCGTGCAGG-3′, 5′-CCGGGCCATGACACTGT-3′), α_x_ integrin (5′-GGTTTCAACTTGGACACAGAGGAGC-3′, 5′-GAAGGGCTGGTGGTAGACGC-3′), and resolved using a 1.5% agarose gel.

### Phagocytosis and efferocytosis assays

Phagocytosis and efferocytosis assays were performed as described previously (Taruc et al., 2018; Yin et al., 2016). Phagocytosis and efferocytosis was quantified by adding 2 µl of IgG or lipid-coated beads per well of macrophages (∼200,000 beads/well, or 800 beads/mm^2^), or adding 5×10^5^ apoptotic cells/well. Cells were centrifuged at 400 ***g*** for 1 min to force beads into contact with macrophages, then incubated at 37°C with 5% CO_2_ for 60 min to allow for phagocytosis and efferocytosis to occur. The cells were then washed three times with cold PBS. Non-internalized IgG beads were labeled using 1:500 dilution of goat anti-human-IgG antibody conjugated to Alexa Fluor 488, non-internalized lipid-coated beads and apoptotic cells were labeled with 1:500 fluorescent streptavidin for 20 min. Cells were then washed three times with PBS, fixed for 15 min with 4% PFA in PBS and mounted in a Leiden chamber for imaging. For quantification, the phagocytic and efferocytic index was calculated using the average number of beads internalized per macrophage, and the Alexa Fluor 488 or Alexa Fluor 647 signal was used to identify the number of bound beads per macrophage. For apoptotic cell uptake, an Otsu threshold was applied to the streptavidin image, the resulting mask inverted, and the inverted mask applied to the cell proliferation dye 670 image to eliminate signal from non-internalized apoptotic cells. The DIC image was then used to manually create polygonal regions of interest (ROIs) around each macrophage, and the integrated cell proliferation dye 670 signal measured for each ROI. The integrated intensity of each cell was then normalized to the average integrated intensity of the control cells, with these averaged values used for comparisons between experimental repeats.

For 3D imaging of efferocytic cups, apoptotic mimics were centrifuged onto macrophages as above and the cells incubated for 30 min at 37°C under 5% CO_2_ to allow for partial engulfment of the beads. The cells were then washed three times with PBS, the membrane labeled with 647-conjugated wheat germ agglutinin (WGA; 1:500) for 10 min at room temperature, then the cells fixed in 4% PFA for 15 min. The cells were then labeled with the desired antibodies (Table S1) as per the immunostaining procedure above. Cells were washed 3×15 min with PBS and stained with secondary antibody diluted 1:1000 in PBS. Z-stacks were acquired using a Zeiss AxioObserver with a 100×/1.40 NA oil objective coupled with a Hamamatsu ORCA-Fusion CMOS camera at a Z-step size of 0.24 μm, then the images deconvolved in Zeiss Zen. FIJI was used to analyze the distribution of the labeled proteins within efferocytic cups, with individual efferocytic cups cropped from the deconvolved *Z*-stack, and a sum-slices projection of the *x*/*z*-axis generated followed by re-slicing of the image to ensure the bottom-to-top direction was aligned along the *x*-axis of the projection. WGA staining was used to identify the top of the cup, with analysis limited to efferocytic cups which engulfed 50–75% of the efferocytic target. The distribution of the WGA staining and labeled signaling molecules throughout the cup was then quantified using the image profile tool, with the intensity of the signaling molecule normalized to WGA (to normalize for membrane density), and the signal scaled 0–1. MERTK cluster size was determined using 3D reconstructions of individual cups and the line tool to measure the diameter across the largest portion of the cluster.

### Integrin activation FRET assay

THP1 cells were plated and differentiated as described above, then cooled to 10°C for 10 min. Cells were washed three times with PBS and incubated with a FITC anti-human CD18 headgroup antibody (clone TS1/18, 0.25 µg/ml) for 20 min at 10°C. This antibody was selected as it does not interfere with integrin conformational changes nor with the formation of molecular complexes that link integrins to actin (Nordenfelt et al., 2016). After 20 min, cells were washed with PBS and incubated with 100 nM octadecyl-rhodamine B (ORB). Unstained, donor-only (FITC-stained) and acceptor-only (ORB-stained) samples were also prepared. Cells were then washed with PBS and pharmacological inhibitors added at the concentrations indicated above. The coverslip was then transferred to a Leiden chamber, and filled with imaging buffer containing Gas6-opsonized apoptotic cell mimics (800 beads/mm^2^), and the chamber transferred to the pre-heated stage of the Leica DMI6000B microscope and imaged for donor fluorescence (Idd, λEx/λEm: 488 nm/525 nm), FRET (Ida, λEx/λEm: 488 nm/578 nm), acceptor fluorescence (Iaa, λEx/λEm: 555 nm/575 nm) and in DIC. As a positive control, 1 mM of Mn^2+^ was added to some samples to force integrins into their active conformation. Images were captured every 2 min for up to an hour. The resulting images were imported into FIJI and corrected sensitized emission FRET calculated using our previously published method (Taefehshokr et al., 2024). Briefly, unstained cells were used to quantify the cellular background in the Idd, Ida and Iaa channels, and these values subtracted from the corresponding channel in all images. Then, donor cross-talk (β, donor-only Ida/Idd), donor cross-excitation (α, acceptor-only Idd/Iaa), acceptor cross-excitation (γ, acceptor-only Ida/Iaa) and FRET cross-talk (δ, acceptor-only Idd/Ida),were calculated. FRET efficiency (E_A_) was then calculated in background subtracted images using the formula:

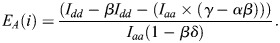


Finally, a mask was generated by applying an Otsu threshold to the Iaa and Idd channel, and the two masks combined using a binary AND command. The resulting mask was then dilated once, and the resulting mask applied to the FRET image to eliminate spurious signals from regions lacking either ORB or β_2_ integrins. To quantify FRET signal within efferocytic cups, an ROI around the efferocytic target was drawn using the DIC channel, and the FRET signal from the masked FRET image quantified within this ROI. This 32-bit image was used to quantify integrin activation, but for images displaying the localization of integrin activation, this image was converted into a 16-bit image, inverted to display integrin activation rather than raw FRET signal and a 16-color ramp LUT applied. The maximum theoretical FRET efficiency of the FITC/ORB FRET pair is 0.3136 (Lambert, 2019).

### Supported lipid bilayer preparation

#1.5 thickness, 18 mm diameter glass coverslips were cleaned in 70% ethanol, distilled water, and then heated to 55°C in 2 M HCl to remove all organic material. These coverslips were then plasma cleaned by a 10 s exposure to air-plasma (<1000 mTorr) in a 1500 W microwave plasma chamber made in house (Tepperman et al., 2020). 0.02 µmol of 100 μm diameter liposomes mimicking either a healthy cell (99.9:0.1 molar ratio of PtdChol:Biotin-PtdEth) or apoptotic cell (79.9:20:0.1 molar ratio of PtdChol:PtdSer:biotin-PtdEth) were prepared as per the manufacturer's instructions and placed on a plasma cleaned coverslip to self-deposit into a supported lipid bilayer (SLB). For some experiments, the biotin-PE was excluded from the lipid mixture, and instead, a small pen mark was placed on the top surface of the coverslip prior to addition of the liposomes. Coverslips were incubated for 25 min, then washed three times with PBS, then incubated for 25 min with 2.5% BSA in PBS with or without 1:1000 strepatavadin–Alexa-Fluor-647 to block any non-lipidated surfaces and to label the monolayer. The SLBs were then washed three times with PBS and placed in a Leiden chamber filled with imaging buffer.

### Efferocytic synapse imaging

∼2.5×10^5^ PMA-differentiated THP1 macrophages were cooled to 10°C, and washed three times with PBS. For FRET imaging, cells were labeled as described above. For synapse imaging, the cells were labeled with 0.25 μg/ml FITC-conjugated mouse-anti-human CD18 antibody plus 0.25 μg/ml rabbit-anti-human-MERTK for 20 min at 10°C, washed three times with PBS and then labeled with a Cy3-labeled anti-rabbit secondary Fab. For single-particle tracking (SPT), cells were labeled as for synapse imaging but using a goat-anti-rabbit-IgG Alexa Fluor 647 secondary Fab in lieu of the Cy3-labeled Fab, producing a label density of ∼1 Cy3 or Alexa Fluor 647 per 2.5 μm^2^. After labeling, the cells were washed three times with PBS and suspended by removing all medium and adding 300 μl of accutase for 10 min at room temperature. 700 μl of FBS-supplemented RPMI were added to each well to suspend the cells, the cell suspension pelleted with a 4 min 400 ***g*** centrifugation, the supernatant was removed, the pellet was resuspended in 800 μl of imaging buffer plus 10% human serum, and the cells added onto Leiden chamber containing an SLB-coated coverslip. For FRET and synapse imaging, 1.6 μm thick *z*-stacks (0.4 μm spacing between slices) were captured of the cells every minute, using the strepatavadin–Alexa Fluor 647-labeled SLB to demark the lower bound of the *z*-stack, and capturing either Idd, Ida, Iaa and DIC channels (FRET imaging) or FITC, Cy3, Alexa Fluor 647 and DIC imaging (synapse imaging). For SPT imaging, the position of the SLB was identified using the pen mark on the coverslip prior to addition of the cell. Every 2 min a single DIC, Alexa Fluor 647 and FITC image was collected, as well as 50 frames at 100 ms/frame of the Cy3 and Alexa Fluor 647 channels for SPT analysis. Integrin activation was quantified using FRET as described above. Colocalization between synapse components was quantified by determining the Pearson correlation as implemented in the Just Another Colocalization Plugin in ImageJ/FIJI (Bolte and Cordelières, 2006; Schindelin et al., 2012). Synapse expansion was quantified by manually tracing the outer edge of the β_2_ ring using the polygon selection tool in ImageJ/FIJI and measuring the Feret's diameter. This measurement was repeated for the cell at each time point, normalizing all diameters to the diameter of the synapse at the first time point a synapse was visible in the time-series. For all analysis, cells were defined as ‘synapsing’ if they were in the same focal plane as the planar lipid bilayer.

### Single-particle tracking

SPT data was analyzed by exporting the time-series of the Cy3 and Alexa Fluor 647 acquisitions as TIFF stacks. Diffusion of MERTK and β_2_ integrin were quantified using the SPT acquisitions as described previously (Goiko et al., 2016, 2018). Briefly, SPT was performed using the MATLAB scripts of Jaqaman et al., and custom-written MATLAB scripts were used to remove any molecular trajectories with a positional accuracy worse than 25 nm (Jaqaman et al., 2008; Goiko et al., 2016). Moment scaling spectrum (MSS) analysis was used to classify the diffusion of MERTK and β_2_ (Ferrari et al., 2001), where MSS classifies molecular movement based on the power-law indices, γ_ρ_, of the first through fourth moments, 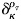, of molecular movement, assuming each moment has a power-law dependency on the lag-time (τ) of 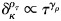, where Brownian motion is defined as ρ≈y_ρ_, subdiffusive (confined) movement as ρ<y_ρ_, and superdiffusive movement as ρ>y_ρ_. The diffusion rate is estimated from the initial slope of mean-squared displacement (MSD) plot of each particle, and for confined particles, the confinement zone diameter is calculated as the 90th percentile of the maximum possible extent of the particles variance-covariance matrix.

### Cluster size analysis

MERTK, α_x_ integrin, β_1_ integrin or β_2_ integrin on THP1-derived macrophages were labeled as described above, using a photosensitive fluorophore (FITC or PE). In some experiments these cells were then fixed with 4% PFA for 20 min at 37°C prior to imaging, whereas for other experiments the macrophages were placed onto supported lipid bilayers containing either 100% PtdChol or 80% PtdChol plus 20% PtdSer for 30 min at 37°C. The cells were imaged with 100 ms exposures and maximum excitation energy (∼2 W/m^2^). Cluster size was then quantified from this time series using the stepwise photobleaching algorithm of Hummert et al. (2021). In our hands, this algorithm was able to reliably detect up to 28 photobleaching steps, so for larger cluster sizes a linear regression was performed on the final 15–20 photobleaching steps to calculate a per-fluorophore integrated intensity, with this value then used to estimate the number of proteins present in larger clusters based on their integrated intensity.

### Statistical analysis

Unless otherwise indicated data are presented as the mean±s.e.m. and analyzed using a two-tailed unpaired Student's *t*-test or one-way ANOVA with Tukey correction. All statistical analyses were performed in GraphPad Prism Version 9 (GraphPad, San Diego, CA, USA).

## Supplementary Material

10.1242/joces.264792_sup1 Supplementary information

## References

[JCS264792C1] Antonieta Cote-Vélez, M. J., Ortega, E. and Ortega, A. (2001). Involvement of pp125FAK and p60SRC in the signaling through Fc gamma RII-Fc gamma RIII in murine macrophages. *Immunol. Lett.* 78, 189-194. 10.1016/S0165-2478(01)00251-6 11578694

[JCS264792C2] Audo, R., Hua, C., Hahne, M., Combe, B., Morel, J. and Daien, C. I. (2017). Phosphatidylserine outer layer translocation is implicated in IL-10 secretion by human regulatory B cells. *PLoS ONE* 12, e0169755. 10.1371/journal.pone.0169755 28072868 PMC5225009

[JCS264792C3] Bain, C. C., Scott, C. L., Uronen-Hansson, H., Gudjonsson, S., Jansson, O., Grip, O., Guilliams, M., Malissen, B., Agace, W. W. and Mowat, A. M. I. (2013). Resident and pro-inflammatory macrophages in the colon represent alternative context-dependent fates of the same Ly6Chi monocyte precursors. *Mucosal Immunol.* 6, 498-510. 10.1038/mi.2012.89 22990622 PMC3629381

[JCS264792C4] Barger, S. R., Vorselen, D., Gauthier, N. C., Theriot, J. A. and Krendel, M. (2022). F-actin organization and target constriction during primary macrophage phagocytosis is balanced by competing activity of myosin-I and myosin-II. *Mol. Biol. Cell* 33, br24. 10.1091/mbc.E22-06-0210 36129777 PMC9727803

[JCS264792C5] Bilsland, C. A. G., Diamond, M. S. and Springer, T. A. (1994). The leukocyte integrin p150,95 (CD11c/CD18) as a receptor for iC3b. Activation by a heterologous beta subunit and localization of a ligand recognition site to the I domain. *J. Immunol.* 152, 4582-4589. 10.4049/jimmunol.152.9.4582 7512600

[JCS264792C6] Binder, M. D., Fox, A. D., Merlo, D., Johnson, L. J., Giuffrida, L., Calvert, S. E., Akkermann, R., Ma, G. Z. M., Perera, A. A., Gresle, M. M. et al. (2016). Common and low frequency variants in MERTK are independently associated with multiple sclerosis susceptibility with discordant association dependent upon HLA-DRB1*15:01 status. *PLoS Genet.* 12, e1005853. 10.1371/journal.pgen.1005853 26990204 PMC4798184

[JCS264792C7] Blackburn, J. W. D., Lau, D. H. C., Liu, E. Y., Ellins, J., Vrieze, A. M., Pawlak, E. N., Dikeakos, J. D. and Heit, B. (2019). Soluble CD93 is an apoptotic cell opsonin recognized by αx β2. *Eur. J. Immunol.* 49, 600-610. 10.1002/eji.201847801 30656676

[JCS264792C8] Bolte, S. and Cordelières, F. P. (2006). A guided tour into subcellular colocalization analysis in light microscopy. *J. Microsc.* 224, 213-232. 10.1111/j.1365-2818.2006.01706.x 17210054

[JCS264792C9] Brea-Fernández, A. J., Pomares, E., Brión, M. J., Marfany, G., Blanco, M. J., Sánchez-Salorio, M., González-Duarte, R. and Carracedo, A. (2008). Novel splice donor site mutation in MERTK gene associated with retinitis pigmentosa. *Br. J. Ophthalmol.* 92, 1419-1423. 10.1136/bjo.2008.139204 18815424

[JCS264792C10] Brouillette, R. B., Phillips, E. K., Patel, R., Mahauad-Fernandez, W., Moller-Tank, S., Rogers, K. J., Dillard, J. A., Cooney, A. L., Martinez-Sobrido, L., Okeoma, C. et al. (2018). TIM-1 mediates dystroglycan-independent entry of lassa virus. *J. Virol.* 92, e00018-e00093. 10.1128/JVI.00093-18 PMC6069209 29875238

[JCS264792C11] Brown, G. D. and Gordon, S. (2001). Immune recognition. A new receptor for beta-glucans. *Nature* 413, 36-37. 10.1038/35092620 11544516

[JCS264792C12] Bruhns, P., Iannascoli, B., England, P., Mancardi, D. A., Fernandez, N., Jorieux, S. and Daëron, M. (2009). Specificity and affinity of human Fcgamma receptors and their polymorphic variants for human IgG subclasses. *Blood* 113, 3716-3725. 10.1182/blood-2008-09-179754 19018092

[JCS264792C13] Caetano, F. A., Dirk, B. S., Tam, J. H. K., Cavanagh, P. C., Goiko, M., Ferguson, S. S. G., Pasternak, S. H., Dikeakos, J. D., De Bruyn, J. R. and Heit, B. (2015). MIiSR: molecular interactions in super-resolution imaging enables the analysis of protein interactions, dynamics and formation of multi-protein structures. *PLoS Comput. Biol.* 11, e1004634. 10.1371/journal.pcbi.1004634 26657340 PMC4676698

[JCS264792C14] Caetano Crowley, F. A., Heit, B. and Ferguson, S. S. G. (2019). Super-resolution imaging of G protein-coupled receptors using ground state depletion microscopy. *Methods Mol. Biol.* 1947, 323-336. 10.1007/978-1-4939-9121-1_18 30969425

[JCS264792C15] Cai, B., Thorp, E. B., Doran, A. C., Sansbury, B. E., Daemen, M. J. A. P., Dorweiler, B., Spite, M., Fredman, G. and Tabas, I. (2017). MerTK receptor cleavage promotes plaque necrosis and defective resolution in atherosclerosis. *J. Clin. Invest.* 127, 1-5. 10.1172/JCI90520 28067670 PMC5272169

[JCS264792C16] Chen, Y., Wang, H., Qi, N., Wu, H., Xiong, W., Ma, J., Lu, Q. and Han, D. (2009). Functions of TAM RTKs in regulating spermatogenesis and male fertility in mice. *Reproduction* 138, 655-666. 10.1530/REP-09-0101 19602523

[JCS264792C17] Chiang, H. Y., Chu, P. H., Chen, S. C. and Lee, T. H. (2021). MFG-E8 regulates vascular smooth muscle cell migration through dose-dependent mediation of actin polymerization. *J. Am. Heart Assoc.* 10, e020870. 10.1161/JAHA.121.020870 34041925 PMC8483510

[JCS264792C18] Chigaev, A., Blenc, A. M., Braaten, J. V., Kumaraswamy, N., Kepley, C. L., Andrews, R. P., Oliver, J. M., Edwards, B. S., Prossnitz, E. R., Larson, R. S. et al. (2001). Real time analysis of the affinity regulation of α4-integrin: the physiologically activated receptor is intermediate in affinity between resting and Mn2+ or antibody activation. *J. Biol. Chem.* 276, 48670-48678. 10.1074/jbc.M103194200 11641394

[JCS264792C19] Choraghe, R. P., Kołodziej, T., Buser, A., Rajfur, Z. and Neumann, A. K. (2020). RHOA-mediated mechanical force generation through Dectin-1. *J. Cell Sci.* 133, jcs236166. 10.1242/jcs.236166 31964711 PMC7063837

[JCS264792C20] Connors, W. L., Jokinen, J., White, D. J., Puranen, J. S., Kankaanpaöaö, P., Upla, P., Tulla, M., Johnson, M. S. and Heino, J. (2007). Two synergistic activation mechanisms of alpha2beta1 integrin-mediated collagen binding. *J. Biol. Chem.* 282, 14675-14683. 10.1074/jbc.M700759200 17374611

[JCS264792C21] Cordoba, S.-P., Choudhuri, K., Zhang, H., Bridge, M., Basat, A. B., Dustin, M. L. and Van Der Merwe, P. A. (2013). The large ectodomains of CD45 and CD148 regulate their segregation from and inhibition of ligated T-cell receptor. *Blood* 121, 4295-4302. 10.1182/blood-2012-07-442251 23580664 PMC3663424

[JCS264792C22] Crowley, M. T., Costello, P. S., Fitzer-Attas, C. J., Turner, M., Meng, F., Lowell, C., Tybulewicz, V. L. J. and DeFranco, A. L. (1997). A critical role for Syk in signal transduction and phagocytosis mediated by Fcgamma receptors on macrophages. *J. Exp. Med.* 186, 1027-1039. 10.1084/jem.186.7.1027 9314552 PMC2199061

[JCS264792C23] Deberge, M., Yeap, X. Y., Dehn, S., Zhang, S., Grigoryeva, L., Misener, S., Procissi, D., Zhou, X., Lee, D. C., Muller, W. A. et al. (2017). MerTK cleavage on resident cardiac macrophages compromises repair after myocardial ischemia reperfusion injury. *Circ. Res.* 121, 930-940. 10.1161/CIRCRESAHA.117.311327 28851810 PMC5623080

[JCS264792C24] Dillon, S. R., Mancini, M., Rosen, A. and Schlissel, M. S. (2000). Annexin V binds to viable B cells and colocalizes with a marker of lipid rafts upon B cell receptor activation. *J. Immunol.* 164, 1322-1332. 10.4049/jimmunol.164.3.1322 10640746

[JCS264792C25] Dransfield, I., Zagórska, A., Lew, E. D., Michail, K. and Lemke, G. (2015). Mer receptor tyrosine kinase mediates both tethering and phagocytosis of apoptotic cells. *Cell Death Dis.* 6, e1646. 10.1038/cddis.2015.18 25695599 PMC4669813

[JCS264792C26] Earl, D. J. and Deem, M. W. (2008). Monte Carlo simulations. *Methods Mol. Biol.* 443, 25-36. 10.1007/978-1-59745-177-2_2 18446280

[JCS264792C27] Elliott, J. I., Surprenant, A., Marelli-Berg, F. M., Cooper, J. C., Cassady-Cain, R. L., Wooding, C., Linton, K., Alexander, D. R. and Higgins, C. F. (2005). Membrane phosphatidylserine distribution as a non-apoptotic signalling mechanism in lymphocytes. *Nat. Cell Biol.* 7, 808-816. 10.1038/ncb1279 16025105

[JCS264792C28] Evans, A. L., Blackburn, J. W. D., Taruc, K., Kipp, A., Dirk, B. S., Hunt, N. R., Barr, S. D., Dikeakos, J. D. and Heit, B. (2017). Antagonistic coevolution of MER tyrosine kinase expression and function. *Mol. Biol. Evol.* 34, 1613-1628. 10.1093/molbev/msx102 28369510 PMC5850725

[JCS264792C29] Feng, W., Yasumura, D., Matthes, M. T., Lavail, M. M. and Vollrath, D. (2002). Mertk triggers uptake of photoreceptor outer segments during phagocytosis by cultured retinal pigment epithelial cells. *J. Biol. Chem.* 277, 17016-17022. 10.1074/jbc.M107876200 11861639

[JCS264792C30] Ferrari, R., Manfroi, A. J. and Young, W. R. (2001). Strongly and weakly self-similar diffusion. *Physica D Nonlinear Phenomena* 154, 111-137. 10.1016/S0167-2789(01)00234-2

[JCS264792C31] Finnemann, S. C. and Nandrot, E. F. (2006). MerTK activation during RPE phagocytosis in vivo requires alphaVbeta5 integrin. *Adv. Exp. Med. Biol.* 572, 499-503. 10.1007/0-387-32442-9_69 17249615 PMC3577060

[JCS264792C32] Fischer, K., Voelkl, S., Berger, J., Andreesen, R., Pomorski, T. and Mackensen, A. (2006). Antigen recognition induces phosphatidylserine exposure on the cell surface of human CD8+ T cells. *Blood* 108, 4094-4101. 10.1182/blood-2006-03-011742 16912227

[JCS264792C33] Flannagan, R. S., Canton, J., Furuya, W., Glogauer, M. and Grinstein, S. (2014). The phosphatidylserine receptor TIM4 utilizes integrins as coreceptors to effect phagocytosis. *Mol. Biol. Cell* 25, 1511-1522. 10.1091/mbc.e13-04-0212 24623723 PMC4004599

[JCS264792C34] Francis, E. A., Xiao, H., Teng, L. H. and Heinrich, V. (2022). Mechanisms of frustrated phagocytic spreading of human neutrophils on antibody-coated surfaces. *Biophys. J.* 121, 4714-4728. 10.1016/j.bpj.2022.10.016 36242516 PMC9748254

[JCS264792C35] Freeman, S. A. and Grinstein, S. (2014). Phagocytosis: receptors, signal integration, and the cytoskeleton. *Immunol. Rev.* 262, 193-215. 10.1111/imr.12212 25319336

[JCS264792C36] Freeman, S. A., Goyette, J., Furuya, W., Woods, E. C., Bertozzi, C. R., Bergmeier, W., Hinz, B., van der Merwe, P. A., Das, R. and Grinstein, S. (2016). Integrins form an expanding diffusional barrier that coordinates phagocytosis. *Cell* 164, 128-140. 10.1016/j.cell.2015.11.048 26771488 PMC4715264

[JCS264792C37] Freeman, S. A., Vega, A., Riedl, M., Collins, R. F., Ostrowski, P. P., Woods, E. C., Bertozzi, C. R., Tammi, M. I., Lidke, D. S., Johnson, P. et al. (2018). Transmembrane pickets connect cyto- and pericellular skeletons forming barriers to receptor engagement. *Cell* 172, 305-312.e10. 10.1016/j.cell.2017.12.023 29328918 PMC5929997

[JCS264792C38] Fricker, M., Neher, J. J., Zhao, J.-W., Théry, C., Tolkovsky, A. M. and Brown, G. C. (2012). MFG-E8 mediates primary phagocytosis of viable neurons during neuroinflammation. *J. Neurosci.* 32, 2657-2666. 10.1523/JNEUROSCI.4837-11.2012 22357850 PMC3312099

[JCS264792C39] Garçon, F. and Okkenhaug, K. (2016). PI3Kδ promotes CD4+ T-cell interactions with antigen-presenting cells by increasing LFA-1 binding to ICAM-1. *Immunol. Cell Biol.* 94, 486-495. 10.1038/icb.2016.1 26740009 PMC4829101

[JCS264792C40] Gardai, S. J., McPhillips, K. A., Frasch, S. C., Janssen, W. J., Starefeldt, A., Murphy-Ullrich, J. E., Bratton, D. L., Oldenborg, P.-A., Michalak, M. and Henson, P. M. (2005). Cell-surface calreticulin initiates clearance of viable or apoptotic cells through trans-activation of LRP on the phagocyte. *Cell* 123, 321-334. 10.1016/j.cell.2005.08.032 16239148

[JCS264792C41] Georgescu, M.-M., Kirsch, K. H., Shishido, T., Zong, C. and Hanafusa, H. (1999). Biological effects of c-Mer receptor tyrosine kinase in hematopoietic cells depend on the Grb2 binding site in the receptor and activation of NF-kappaB. *Mol. Cell. Biol.* 19, 1171-1181. 10.1128/MCB.19.2.1171 9891051 PMC116046

[JCS264792C42] Goiko, M., de Bruyn, J. R. and Heit, B. (2016). Short-lived cages restrict protein diffusion in the plasma membrane. *Sci. Rep.* 6, 34987. 10.1038/srep34987 27725698 PMC5057110

[JCS264792C43] Goiko, M., De Bruyn, J. R. and Heit, B. (2018). Membrane diffusion occurs by continuous-time random walk sustained by vesicular trafficking. *Biophys. J.* 114, 2887-2899. 10.1016/j.bpj.2018.04.024 29925025 PMC6026331

[JCS264792C44] Heit, B., Tavener, S., Raharjo, E. and Kubes, P. (2002). An intracellular signaling hierarchy determines direction of migration in opposing chemotactic gradients. *J. Cell Biol.* 159, 91-102. 10.1083/jcb.200202114 12370241 PMC2173486

[JCS264792C45] Heit, B., Robbins, S. M., Downey, C. M., Guan, Z., Colarusso, P., Miller, B. J., Jirik, F. R. and Kubes, P. (2008). PTEN functions to “prioritize” chemotactic cues and prevent “distraction” in migrating neutrophils. *Nat. Immunol.* 9, 743-752. 10.1038/ni.1623 18536720

[JCS264792C46] Heit, B., Kim, H., Cosío, G., Castaño, D., Collins, R., Lowell, C. A., Kain, K. C., Trimble, W. S. and Grinstein, S. (2013). Multimolecular signaling complexes enable Syk-mediated signaling of CD36 internalization. *Dev. Cell* 24, 372-383. 10.1016/j.devcel.2013.01.007 23395392 PMC3586299

[JCS264792C47] Hirano, Y., Yang, W.-L., Aziz, M., Zhang, F., Sherry, B. and Wang, P. (2017). MFG-E8-derived peptide attenuates adhesion and migration of immune cells to endothelial cells. *J. Leukoc. Biol.* 101, 1201-1209. 10.1189/jlb.3A0416-184RR 28096298 PMC5380373

[JCS264792C48] Hu, B., Jennings, J. H., Sonstein, J., Floros, J., Todt, J. C., Polak, T. and Curtis, J. L. (2004). Resident murine alveolar and peritoneal macrophages differ in adhesion of apoptotic thymocytes. *Am. J. Respir. Cell Mol. Biol.* 30, 687-693. 10.1165/rcmb.2003-0255OC 14527926 PMC4138126

[JCS264792C49] Hummert, J., Yserentant, K., Fink, T., Euchner, J., Ho, Y. X., Tashev, S. A. and Herten, D.-P. (2021). Photobleaching step analysis for robust determination of protein complex stoichiometries. *Mol. Biol. Cell* 32, ar35. 10.1091/mbc.E20-09-0568 34586828 PMC8693960

[JCS264792C50] Hurtado, B. B. B., Abasolo, N., Muñoz, X., García, N., Benavente, Y., Rubio, F., García de Frutos, P., Krupinski, J. and Sala, N. N. (2010). Association study between polymorphims in GAS6-TAM genes and carotid atherosclerosis. *Thromb. Haemostasis* 104, 592-598. 10.1160/TH09-11-0787 20664904

[JCS264792C51] Jaqaman, K., Loerke, D., Mettlen, M., Kuwata, H., Grinstein, S., Schmid, S. L. and Danuser, G. (2008). Robust single-particle tracking in live-cell time-lapse sequences. *Nat. Methods* 5, 695-702. 10.1038/nmeth.1237 18641657 PMC2747604

[JCS264792C52] Jaqaman, K., Kuwata, H., Touret, N., Collins, R., Trimble, W. S., Danuser, G. and Grinstein, S. (2011). Cytoskeletal control of CD36 diffusion promotes its receptor and signaling function. *Cell* 146, 593-606. 10.1016/j.cell.2011.06.049 21854984 PMC3160624

[JCS264792C53] Jaumouillé, V., Cartagena-Rivera, A. X. and Waterman, C. M. (2019). Coupling of β2 integrins to actin by a mechanosensitive molecular clutch drives complement receptor-mediated phagocytosis. *Nat. Cell Biol.* 21, 1357-1369. 10.1038/s41556-019-0414-2 31659275 PMC6858589

[JCS264792C54] Jensen, R. K., Bajic, G., Sen, M., Springer, T. A., Vorup-Jensen, T. and Andersen, G. R. (2021). Complement receptor 3 forms a compact high-affinity complex with iC3b. *J. Immunol.* 206, 3032-3042. 10.4049/jimmunol.2001208 34117107

[JCS264792C55] Jiang, Y., Zhang, Y., Leung, J. Y., Fan, C., Popov, K. I., Su, S., Qian, J., Wang, X., Holtzhausen, A., Ubil, E. et al. (2019). MERTK mediated novel site Akt phosphorylation alleviates SAV1 suppression. *Nat. Commun.* 10, 1515. 10.1038/s41467-019-09233-7 30944303 PMC6447540

[JCS264792C56] Kiefer, F., Brumell, J., Al-Alawi, N., Latour, S., Cheng, A., Veillette, A., Grinstein, S. and Pawson, T. (1998). The Syk protein tyrosine kinase is essential for Fcgamma receptor signaling in macrophages and neutrophils. *Mol. Cell. Biol.* 18, 4209-4220. 10.1128/MCB.18.7.4209 9632805 PMC109005

[JCS264792C57] Klapholz, B. and Brown, N. H. (2017). Talin - the master of integrin adhesions. *J. Cell Sci.* 130, 2435-2446. 10.1242/jcs.190991 28701514

[JCS264792C58] Kovari, D. T., Wei, W., Chang, P., Toro, J.-S., Beach, R. F., Chambers, D., Porter, K., Koo, D. and Curtis, J. E. (2016). Frustrated phagocytic spreading of J774A-1 macrophages ends in myosin ii-dependent contraction. *Biophys. J.* 111, 2698-2710. 10.1016/j.bpj.2016.11.009 28002746 PMC5194617

[JCS264792C59] Kusumi, A., Nakada, C., Ritchie, K., Murase, K., Suzuki, K., Murakoshi, H., Kasai, R. S., Kondo, J. and Fujiwara, T. (2005). Paradigm shift of the plasma membrane concept from the two-dimensional continuum fluid to the partitioned fluid: high-speed single-molecule tracking of membrane molecules. *Annu. Rev. Biophys. Biomol. Struct.* 34, 351-378. 10.1146/annurev.biophys.34.040204.144637 15869394

[JCS264792C60] Laine, R. F., Heil, H. S., Coelho, S., Nixon-Abell, J., Jimenez, A., Wiesner, T., Martínez, D., Galgani, T., Régnier, L., Stubb, A. et al. (2023). High-fidelity 3D live-cell nanoscopy through data-driven enhanced super-resolution radial fluctuation. *Nat. Methods* 20, 1949-1956. 10.1038/s41592-023-02057-w 37957430 PMC10703683

[JCS264792C61] Lam, A. L. and Heit, B. (2021). Having an old friend for dinner: the interplay between apoptotic cells and efferocytes. *Cells* 10, 1265. 10.3390/cells10051265 34065321 PMC8161178

[JCS264792C62] Lambert, T. J. (2019). FPbase: a community-editable fluorescent protein database. *Nat. Methods* 16, 277-278. 10.1038/s41592-019-0352-8 30886412

[JCS264792C63] Law, A.-L., Parinot, C., Chatagnon, J., Gravez, B., Sahel, J.-A., Bhattacharya, S. S. and Nandrot, E. F. (2015). Cleavage of Mer tyrosine kinase (MerTK) from the cell surface contributes to the regulation of retinal phagocytosis. *J. Biol. Chem.* 290, 4941-4952. 10.1074/jbc.M114.628297 25538233 PMC4335232

[JCS264792C64] Lee, W. L., Cosio, G., Ireton, K. and Grinstein, S. (2007). Role of CrkII in Fcgamma receptor-mediated phagocytosis. *J. Biol. Chem.* 282, 11135-11143. 10.1074/jbc.M700823200 17308335

[JCS264792C65] Lefort, C. T., Hyun, Y.-M., Schultz, J. B., Law, F.-Y., Waugh, R. E., Knauf, P. A. and Kim, M. (2009). Outside-in signal transmission by conformational changes in integrin Mac-1. *J. Immunol.* 183, 6460-6468. 10.4049/jimmunol.0900983 19864611 PMC2860599

[JCS264792C66] Lentz, B. R. (2003). Exposure of platelet membrane phosphatidylserine regulates blood coagulation. *Prog. Lipid Res.* 42, 423-438. 10.1016/S0163-7827(03)00025-0 12814644

[JCS264792C67] Leventis, P. A. and Grinstein, S. (2010). The distribution and function of phosphatidylserine in cellular membranes. *Annu. Rev. Biophys.* 39, 407-427. 10.1146/annurev.biophys.093008.131234 20192774

[JCS264792C68] Li, J., Yan, J. and Springer, T. A. (2021). Low-affinity integrin states have faster ligand-binding kinetics than the high-affinity state. *eLife* 10, e73359. 10.7554/eLife.73359 34854380 PMC8730728

[JCS264792C69] Lin, J., Kurilova, S., Scott, B. L., Bosworth, E., Iverson, B. E., Bailey, E. M. and Hoppe, A. D. (2016). TIRF imaging of Fc gamma receptor microclusters dynamics and signaling on macrophages during frustrated phagocytosis. *BMC Immunol.* 17, 5. 10.1186/s12865-016-0143-2 26970734 PMC4789268

[JCS264792C70] Lopes, F. B., Bálint, Š., Valvo, S., Felce, J. H., Hessel, E. M., Dustin, M. L. and Davis, D. M. (2017). Membrane nanoclusters of FcγRI segregate from inhibitory SIRPα upon activation of human macrophages. *J. Cell Biol.* 216, 1123-1141. 10.1083/jcb.201608094 28289091 PMC5379948

[JCS264792C71] Lukácsi, S., Gerecsei, T., Balázs, K., Francz, B., Szabó, B., Erdei, A. and Bajtay, Z. (2020). The differential role of CR3 (CD11b/CD18) and CR4 (CD11c/CD18) in the adherence, migration and podosome formation of human macrophages and dendritic cells under inflammatory conditions. *PLoS ONE* 15, e0232432. 10.1371/journal.pone.0232432 32365067 PMC7197861

[JCS264792C72] Lv, Z., Bian, Z., Shi, L., Niu, S., Ha, B., Tremblay, A., Li, L., Zhang, X., Paluszynski, J., Liu, M. et al. (2015). Loss of cell surface CD47 clustering formation and binding avidity to SIRPα facilitate apoptotic cell clearance by macrophages. *J. Immunol.* 195, 661-671. 10.4049/jimmunol.1401719 26085683 PMC4490976

[JCS264792C73] Ma, G. Z. M., Stankovich, J., Kilpatrick, T. J., Binder, M. D. and Field, J. (2011). Polymorphisms in the receptor tyrosine kinase MERTK gene are associated with multiple sclerosis susceptibility. *PLoS ONE* 6, e16964. 10.1371/journal.pone.0016964 21347448 PMC3035668

[JCS264792C74] Mao, Y. and Finnemann, S. C. (2012). Essential diurnal Rac1 activation during retinal phagocytosis requires αvβ5 integrin but not tyrosine kinases focal adhesion kinase or Mer tyrosine kinase. *Mol. Biol. Cell* 23, 1104-1114. 10.1091/mbc.e11-10-0840 22262456 PMC3302737

[JCS264792C75] Marchetti, L., Callegari, A., Luin, S., Signore, G., Viegi, A., Beltram, F. and Cattaneo, A. (2013). Ligand signature in the membrane dynamics of single TrkA receptor molecules. *J. Cell Sci.* 126, 4445-4456. 10.1242/jcs.129916 23886941

[JCS264792C76] McDowall, A., Leitinger, B., Stanley, P., Bates, P. A., Randi, A. M. and Hogg, N. (1998). The I domain of integrin leukocyte function-associated antigen-1 is involved in a conformational change leading to high affinity binding to ligand intercellular adhesion molecule 1 (ICAM-1). *J. Biol. Chem.* 273, 27396-27403. 10.1074/jbc.273.42.27396 9765268

[JCS264792C77] Metropolis, N. and Ulam, S. (1949). The Monte Carlo Method. *J. Am. Stat. Assoc.* 44, 335-341. 10.1080/01621459.1949.10483310 18139350

[JCS264792C78] Moon, B., Lee, J., Lee, S.-A., Min, C., Moon, H., Kim, D., Yang, S., Moon, H., Jeon, J., Joo, Y.-E. et al. (2020). Mertk interacts with Tim-4 to enhance Tim-4-mediated efferocytosis. *Cells* 9, E1625. 10.3390/cells9071625 PMC7408610 32640697

[JCS264792C79] Morrissey, M. A., Kern, N. and Vale, R. D. (2020). CD47 ligation repositions the inhibitory receptor SIRPA to suppress integrin activation and phagocytosis. *Immunity* 53, 290-302.e6. 10.1016/j.immuni.2020.07.008 32768386 PMC7453839

[JCS264792C80] Nagy-Baló, Z., Kiss, R., Menge, A., Bödör, C., Bajtay, Z. and Erdei, A. (2020). Activated human memory B lymphocytes use CR4 (CD11c/CD18) for adhesion, migration, and proliferation. *Front. Immunol.* 11, 565458. 10.3389/fimmu.2020.565458 33133077 PMC7550640

[JCS264792C81] Nandrot, E. F., Silva, K. E., Scelfo, C. and Finnemann, S. C. (2012). Retinal pigment epithelial cells use a MerTK-dependent mechanism to limit the phagocytic particle binding activity of αvβ5 integrin. *Biol. Cell* 104, 326-341. 10.1111/boc.201100076 22289110 PMC3577091

[JCS264792C82] Nishi, C., Toda, S., Segawa, K. and Nagata, S. (2014). Tim4- and MerTK-mediated engulfment of apoptotic cells by mouse resident peritoneal macrophages. *Mol. Cell. Biol.* 34, 1512-1520. 10.1128/MCB.01394-13 24515440 PMC3993587

[JCS264792C83] Nordenfelt, P., Elliott, H. L. and Springer, T. A. (2016). Coordinated integrin activation by actin-dependent force during T-cell migration. *Nat. Commun.* 7, 13119. 10.1038/ncomms13119 27721490 PMC5062559

[JCS264792C84] Park, D., Tosello-Trampont, A.-C., Elliott, M. R., Lu, M., Haney, L. B., Ma, Z., Klibanov, A. L., Mandell, J. W. and Ravichandran, K. S. (2007). BAI1 is an engulfment receptor for apoptotic cells upstream of the ELMO/Dock180/Rac module. *Nature* 450, 430-434. 10.1038/nature06329 17960134

[JCS264792C85] Penberthy, K. K., Rival, C., Shankman, L. S., Raymond, M. H., Zhang, J., Perry, J. S. A., Lee, C. S., Han, C. Z., Onengut-Gumuscu, S., Palczewski, K. et al. (2017). Context-dependent compensation among phosphatidylserine-recognition receptors. *Sci. Rep.* 7, 14623. 10.1038/s41598-017-15191-1 29116131 PMC5676788

[JCS264792C86] Pereira, P. M., Albrecht, D., Culley, S., Jacobs, C., Marsh, M., Mercer, J. and Henriques, R. (2019). Fix your membrane receptor imaging: actin cytoskeleton and CD4 membrane organization disruption by chemical fixation. *Front. Immunol.* 10, 675. 10.3389/fimmu.2019.00675 31024536 PMC6460894

[JCS264792C87] Rose, M., Hirmiz, N., Moran-Mirabal, J. M. and Fradin, C. (2015). Lipid diffusion in supported lipid bilayers: a comparison between line-scanning fluorescence correlation spectroscopy and single-particle tracking. *Membranes* 5, 702-721. 10.3390/membranes5040702 26610279 PMC4704007

[JCS264792C88] Sadhu, C., Ting, H. J., Lipsky, B., Hensley, K., Garcia-Martinez, L. F., Simon, S. I. and Staunton, D. E. (2007). CD11c/CD18: novel ligands and a role in delayed-type hypersensitivity. *J. Leukoc. Biol.* 81, 1395-1403. 10.1189/jlb.1106680 17389580

[JCS264792C89] Sándor, N., Lukácsi, S., Ungai-Salánki, R., Orgován, N., Szabó, B., Horváth, R., Erdei, A. and Bajtay, Z. (2016). CD11c/CD18 dominates adhesion of human monocytes, macrophages and dendritic cells over CD11b/CD18. *PLoS ONE* 11, e0163120. 10.1371/journal.pone.0163120 27658051 PMC5033469

[JCS264792C90] Sayedyahossein, S., Nini, L., Irvine, T. S. and Dagnino, L. (2012). Essential role of integrin-linked kinase in regulation of phagocytosis in keratinocytes. *FASEB J.* 26, 4218-4229. 10.1096/fj.12-207852 22767228

[JCS264792C91] Schindelin, J., Arganda-Carreras, I., Frise, E., Kaynig, V., Longair, M., Pietzsch, T., Preibisch, S., Rueden, C., Saalfeld, S., Schmid, B. et al. (2012). Fiji: an open-source platform for biological-image analysis. *Nat. Methods* 9, 676-682. 10.1038/nmeth.2019 22743772 PMC3855844

[JCS264792C92] Schwartz, M. A. (2011). Super-resolution microscopy: a new dimension in focal adhesions. *Curr. Biol.* 21, R115-R116. 10.1016/j.cub.2010.12.025 21300274

[JCS264792C93] Segawa, K., Kurata, S., Yanagihashi, Y., Brummelkamp, T. R., Matsuda, F. and Nagata, S. (2014). Caspase-mediated cleavage of phospholipid flippase for apoptotic phosphatidylserine exposure. *Science* 344, 1164-1168. 10.1126/science.1252809 24904167

[JCS264792C94] Seitz, H. M., Camenisch, T. D., Lemke, G., Earp, H. S. and Matsushima, G. K. (2007). Macrophages and dendritic cells use different Axl/Mertk/Tyro3 receptors in clearance of apoptotic cells. *J. Immunol.* 178, 5635-5642. 10.4049/jimmunol.178.9.5635 17442946

[JCS264792C95] Shelby, S. J., Colwill, K., Dhe-Paganon, S., Pawson, T. and Thompson, D. A. (2013). MERTK interactions with SH2-domain proteins in the retinal pigment epithelium. *PLoS ONE* 8, e53964. 10.1371/journal.pone.0053964 23390493 PMC3563642

[JCS264792C96] Shiratsuchi, H. and Basson, M. D. (2004). Extracellular pressure stimulates macrophage phagocytosis by inhibiting a pathway involving FAK and ERK. *Am. J. Physiol. Cell Physiol.* 286, C1358-C1366. 10.1152/ajpcell.00553.2003 14761895

[JCS264792C97] Smith, A. L., Friedman, D. B., Yu, H., Carnahan, R. H. and Reynolds, A. B. (2011). ReCLIP (reversible cross-link immuno-precipitation): an efficient method for interrogation of labile protein complexes. *PLoS ONE* 6, e16206. 10.1371/journal.pone.0016206 21283770 PMC3024417

[JCS264792C98] Smrž, D., Dráberová, L. and Dráber, P. (2007). Non-apoptotic phosphatidylserine externalization induced by engagement of glycosylphosphatidylinositol-anchored proteins. *J. Biol. Chem.* 282, 10487-10497. 10.1074/jbc.M611090200 17284440

[JCS264792C99] Stowell, S. R., Karmakar, S., Stowell, C. J., Dias-Baruffi, M., McEver, R. P. and Cummings, R. D. (2007). Human galectin-1, -2, and -4 induce surface exposure of phosphatidylserine in activated human neutrophils but not in activated T cells. *Blood* 109, 219-227. 10.1182/blood-2006-03-007153 16940423 PMC1785076

[JCS264792C100] Su, Z., Dhusia, K. and Wu, Y. (2021). A multiscale study on the mechanisms of spatial organization in ligand-receptor interactions on cell surfaces. *Comput. Struct. Biotechnol. J.* 19, 1620-1634. 10.1016/j.csbj.2021.03.024 33868599 PMC8026753

[JCS264792C101] Sugiyama, M. G., Brown, A. I., Vega-Lugo, J., Borges, J. P., Scott, A. M., Jaqaman, K., Fairn, G. D. and Antonescu, C. N. (2023). Confinement of unliganded EGFR by tetraspanin nanodomains gates EGFR ligand binding and signaling. *Nat. Commun.* 14, 2681. 10.1038/s41467-023-38390-z 37160944 PMC10170156

[JCS264792C102] Suzuki, J., Imanishi, E. and Nagata, S. (2016). Xkr8 phospholipid scrambling complex in apoptotic phosphatidylserine exposure. *Proc. Natl. Acad. Sci. USA* 113, 9509-9514. 10.1073/pnas.1610403113 27503893 PMC5003272

[JCS264792C103] Taefehshokr, N., Lac, A., Vrieze, A. M., Dickson, B. H., Guo, P. N., Jung, C., Blythe, E. N., Fink, C., Aktar, A., Dikeakos, J. D. et al. (2024). SARS-CoV-2 NSP5 antagonizes MHC II expression by subverting histone deacetylase 2. *J. Cell Sci.* 137, jcs262172. 10.1242/jcs.262172 38682259 PMC11166459

[JCS264792C104] Taruc, K., Yin, C., Wootton, D. G. and Heit, B. (2018). Quantification of efferocytosis by single-cell fluorescence microscopy. *J. Vis. Exp.* 138, 58149. 10.3791/58149 PMC6128118 30176011

[JCS264792C105] Tepperman, A., Zheng, D. J., Taka, M. A., Vrieze, A., Le Lam, A. and Heit, B. (2020). Customizable live-cell imaging chambers for multimodal and multiplex fluorescence microscopy. *Biochem. Cell Biol.* 98, 612-623. 10.1139/bcb-2020-0064 32339465

[JCS264792C124] Thamilselvan, V., Craig, D. H. and Basson, M. D. (2007). FAK association with multiple signal proteins mediates pressure-induced colon cancer cell adhesion via a Src-dependent PI3K/Akt pathway. *FASEB J.* 21, 1730-1741. 10.1096/fj.06-6545com 17317726

[JCS264792C106] Thiagarajan, P. and Tait, J. F. (1990). Binding of annexin V/placental anticoagulant protein I to platelets. Evidence for phosphatidylserine exposure in the procoagulant response of activated platelets. *J. Biol. Chem.* 265, 17420-17423. 10.1016/S0021-9258(18)38177-8 2145274

[JCS264792C107] Thorp, E., Cui, D., Schrijvers, D. M., Kuriakose, G. and Tabas, I. (2008). Mertk receptor mutation reduces efferocytosis efficiency and promotes apoptotic cell accumulation and plaque necrosis in atherosclerotic lesions of apoe−/− mice. *Arterioscler. Thromb. Vasc. Biol.* 28, 1421-1428. 10.1161/ATVBAHA.108.167197 18451332 PMC2575060

[JCS264792C108] Toda, S., Hanayama, R. and Nagata, S. (2012). Two-step engulfment of apoptotic cells. *Mol. Cell. Biol.* 32, 118-125. 10.1128/MCB.05993-11 22037761 PMC3255703

[JCS264792C109] Toda, S., Segawa, K. and Nagata, S. (2014). MerTK-mediated engulfment of pyrenocytes by central macrophages in erythroblastic islands. *Blood* 123, 3963-3971. 10.1182/blood-2014-01-547976 24659633

[JCS264792C110] Torres-Gomez, A., Sanchez-Trincado, J. L., Toribio, V., Torres-Ruiz, R., Rodríguez-Perales, S., Yáñez-Mó, M., Reche, P. A., Cabañas, C. and Lafuente, E. M. (2020). RIAM-VASP module relays integrin complement receptors in outside-in signaling driving particle engulfment. *Cells* 9, E1166. 10.3390/cells9051166 PMC7291270 32397169

[JCS264792C111] Tsou, W.-I., Nguyen, K.-Q. N., Calarese, D. A., Garforth, S. J., Antes, A. L., Smirnov, S. V., Almo, S. C., Birge, R. B. and Kotenko, S. V. (2014). Receptor tyrosine kinases, TYRO3, AXL, and MER, demonstrate distinct patterns and complex regulation of ligand-induced activation. *J. Biol. Chem.* 289, 25750-25763. 10.1074/jbc.M114.569020 25074926 PMC4162177

[JCS264792C112] Vachon, E., Martin, R., Kwok, V., Cherepanov, V., Chow, C.-W., Doerschuk, C. M., Plumb, J., Grinstein, S. and Downey, G. P. (2007). CD44-mediated phagocytosis induces inside-out activation of complement receptor-3 in murine macrophages. *Blood* 110, 4492-4502. 10.1182/blood-2007-02-076539 17827392 PMC2234794

[JCS264792C113] van den Eijnde, S. M., van den Hoff, M. J. B., Reutelingsperger, C. P. M., van Heerde, W. L., Henfling, M. E. R., Vermeij-Keers, C., Schutte, B., Borgers, M. and Ramaekers, F. C. S. (2001). Transient expression of phosphatidylserine at cell-cell contact areas is required for myotube formation. *J. Cell Sci.* 114, 3631-3642. 10.1242/jcs.114.20.3631 11707515

[JCS264792C114] van der Meer, J. H. M., van der Poll, T. and van ‘t Veer, C. (2014). TAM receptors, Gas6, and protein S: roles in inflammation and hemostasis. *Blood* 123, 2460-2469. 10.1182/blood-2013-09-528752 24596417

[JCS264792C115] Vernon-Wilson, E. F., Kee, W.-J., Willis, A. C., Barclay, A. N., Simmons, D. L. and Brown, M. H. (2000). CD47 is a ligand for rat macrophage membrane signal regulatory protein SIRP (OX41) and human SIRPalpha 1. *Eur. J. Immunol.* 30, 2130-2137. 10.1002/1521-4141(2000)30:8<2130::AID-IMMU2130>3.0.CO;2-8 10940903

[JCS264792C116] Vorselen, D., Barger, S. R., Wang, Y., Cai, W., Theriot, J. A., Gauthier, N. C. and Krendel, M. (2021). Phagocytic “teeth” and myosin-II “jaw” power target constriction during phagocytosis. *eLife* 10, e68627. 10.7554/eLife.68627 34708690 PMC8585483

[JCS264792C117] Walbaum, S., Ambrosy, B., Schütz, P., Bachg, A. C., Horsthemke, M., Leusen, J. H. W., Mócsai, A. and Hanley, P. J. (2021). Complement receptor 3 mediates both sinking phagocytosis and phagocytic cup formation via distinct mechanisms. *J. Biol. Chem.* 296, 100256. 10.1016/j.jbc.2021.100256 33839682 PMC7948798

[JCS264792C118] Wong, H. S., Jaumouillé, V., Heit, B., Doodnauth, S. A., Patel, S., Huang, Y.-W., Grinstein, S. and Robinson, L. A. (2014). Cytoskeletal confinement of CX3CL1 limits its susceptibility to proteolytic cleavage by ADAM10. *Mol. Biol. Cell* 25, 3884-3899. 10.1091/mbc.e13-11-0633 25253723 PMC4244198

[JCS264792C119] Wu, Y., Singh, S., Georgescu, M.-M. and Birge, R. B. (2005). A role for Mer tyrosine kinase in alphavbeta5 integrin-mediated phagocytosis of apoptotic cells. *J. Cell Sci.* 118, 539-553. 10.1242/jcs.01632 15673687

[JCS264792C120] Xu, S., Wang, J., Wang, J.-H. and Springer, T. A. (2017). Distinct recognition of complement iC3b by integrins αXβ2 and αMβ2. *Proc. Natl. Acad. Sci. USA* 114, 3403-3408. 10.1073/pnas.1620881114 28292891 PMC5380021

[JCS264792C121] Yin, C., Kim, Y., Argintaru, D. and Heit, B. (2016). Rab17 mediates differential antigen sorting following efferocytosis and phagocytosis. *Cell Death Dis.* 7, e2529. 10.1038/cddis.2016.431 28005073 PMC5261003

[JCS264792C122] Yin, C., Vrieze, A. M., Rosoga, M., Akingbasote, J., Pawlak, E. N., Jacob, R. A., Hu, J., Sharma, N., Dikeakos, J. D., Barra, L. et al. (2020). Efferocytic defects in early atherosclerosis are driven by GATA2 overexpression in macrophages. *Front. Immunol.* 11, 594136. 10.3389/fimmu.2020.594136 33193444 PMC7644460

[JCS264792C123] Zhang, W., Zhang, D., Stashko, M. A., Deryckere, D., Hunter, D., Kireev, D., Miley, M. J., Cummings, C., Lee, M., Norris-Drouin, J. et al. (2013). Pseudo-cyclization through intramolecular hydrogen bond enables discovery of pyridine substituted pyrimidines as new mer kinase inhibitors. *J. Med. Chem.* 56, 9683-9692. 10.1021/jm401387j 24195762 PMC3980660

