# Supplementary material for: MERTK coordinates efferocytosis by regulating integrin localization and activation
Source: J Cell Sci. 2026 Mar 25;139(6):jcs264792. doi: 10.1242/jcs.264792 (PMC13070255; doi:10.1242/jcs.264792)
Supplement: Supplementary information [file joces-139-264792-s1.pdf]

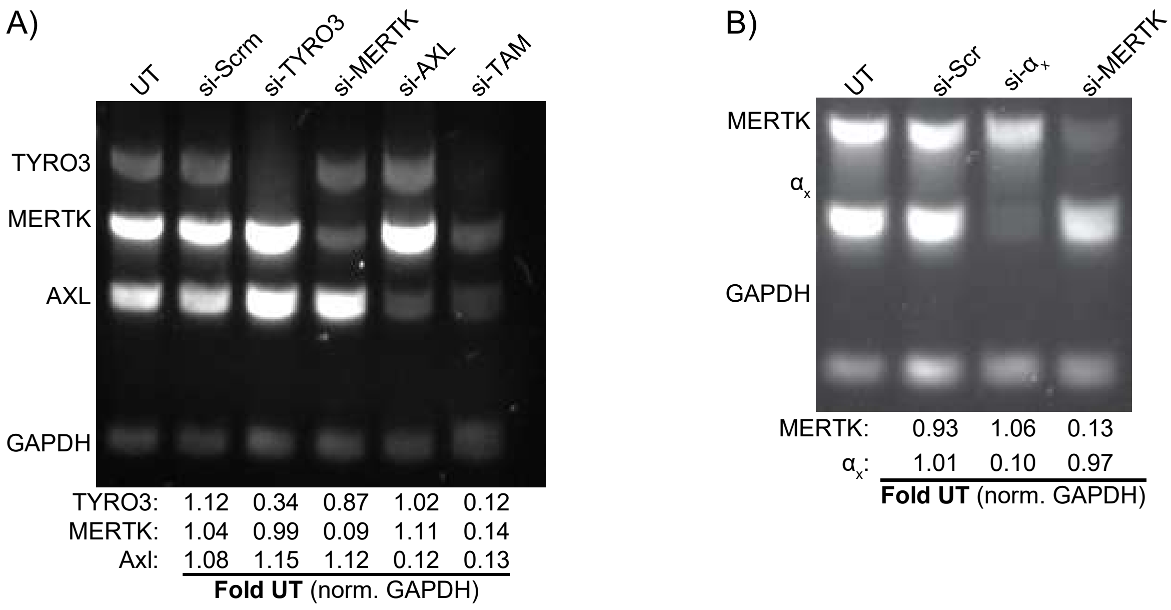


**Fig. S1.** Confirmation of siRNA depletion by semi-quantitative RT-PCR. **A)** Depletion of the TAM receptors in THP1-macrophages, with receptors depleted individually (si-MERTK, si-Axl and si-Tyro3) and combined (si-TAM). **B)** siRNA knockdown in THP1-macrophages of MERTK and $\alpha_x$ integrin. UT = cells not treated with siRNA, si-Scrm = cells treated with a scrambled (non-targeting) siRNA. Data is representative of 3 independent experiments.

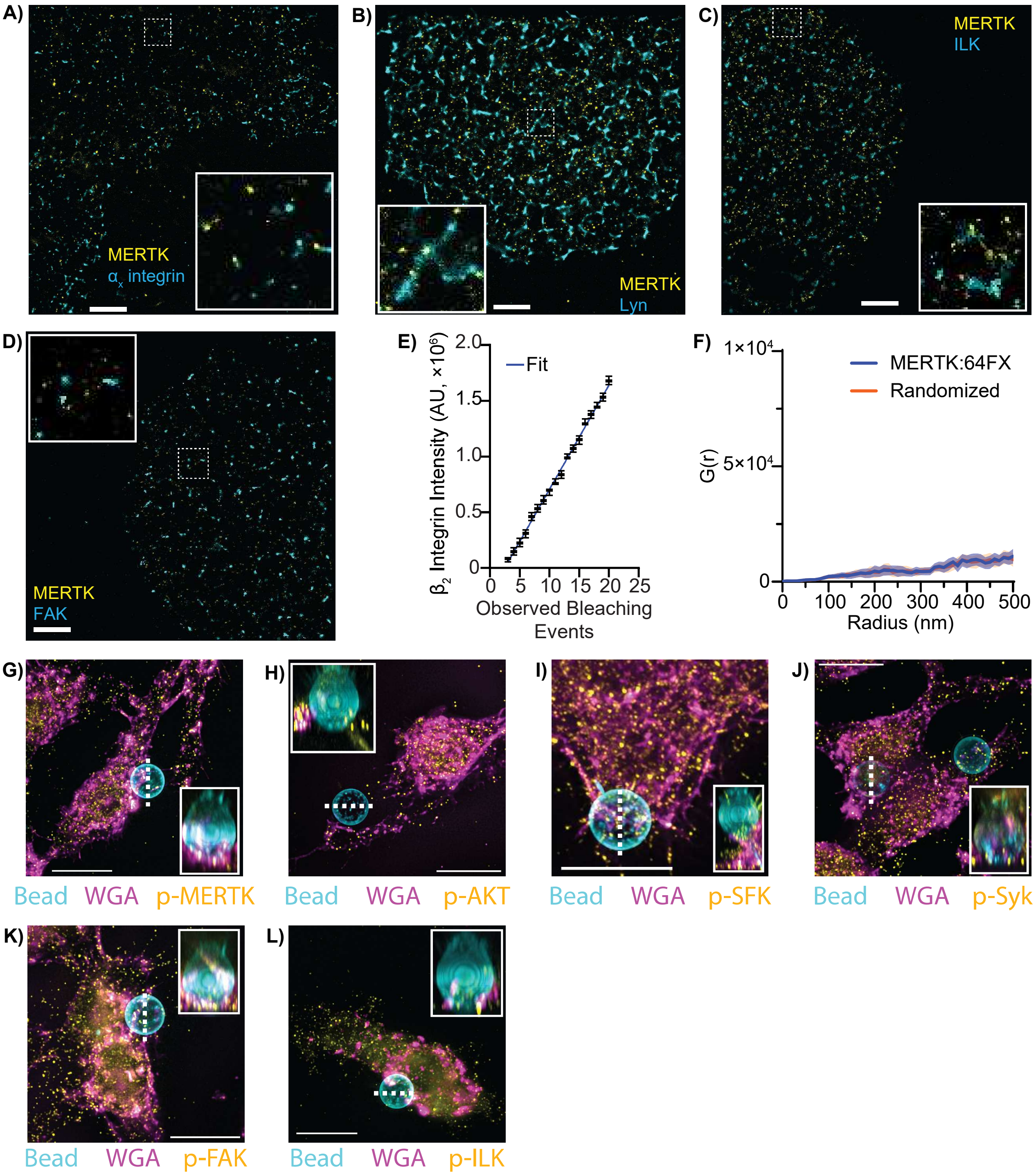


**Fig. S2. A-D)** Ground state depletion microscopy images showing the distribution on the surface of macrophages of MERTK (yellow) and $\alpha_x$ integrin (A), Lyn (B), ILK (C), and FAK (D). Inserts show regions within the dotted boxes, scale bars are 2 µm. **E)** Linear regression analysis of step-wise photobleacing to determine the integrated intensity of individual $\beta_2$ integrins. **F)** Radial distribution analysis of MERTK with the fixable membrane dye 64FX. **G-L)** Maximum intensity projections of macrophages with partically engulfed apoptotic mimics. Macrophages have been stained with WGA (magenta) and antibodies specific to the phos-phorylated (activated) forms of MERTK (G), AKT (H), Src-family kinases (SFK, I), Syk (J), FAK (K), and ILK (L). Inserts show a maximum intensity projection of the efferocytic cup, with the projection made along the dotted line, scale bars are 10 µm. Data is representative of 3-5 independent experiments.

Journal of Cell Science • Supplementary information

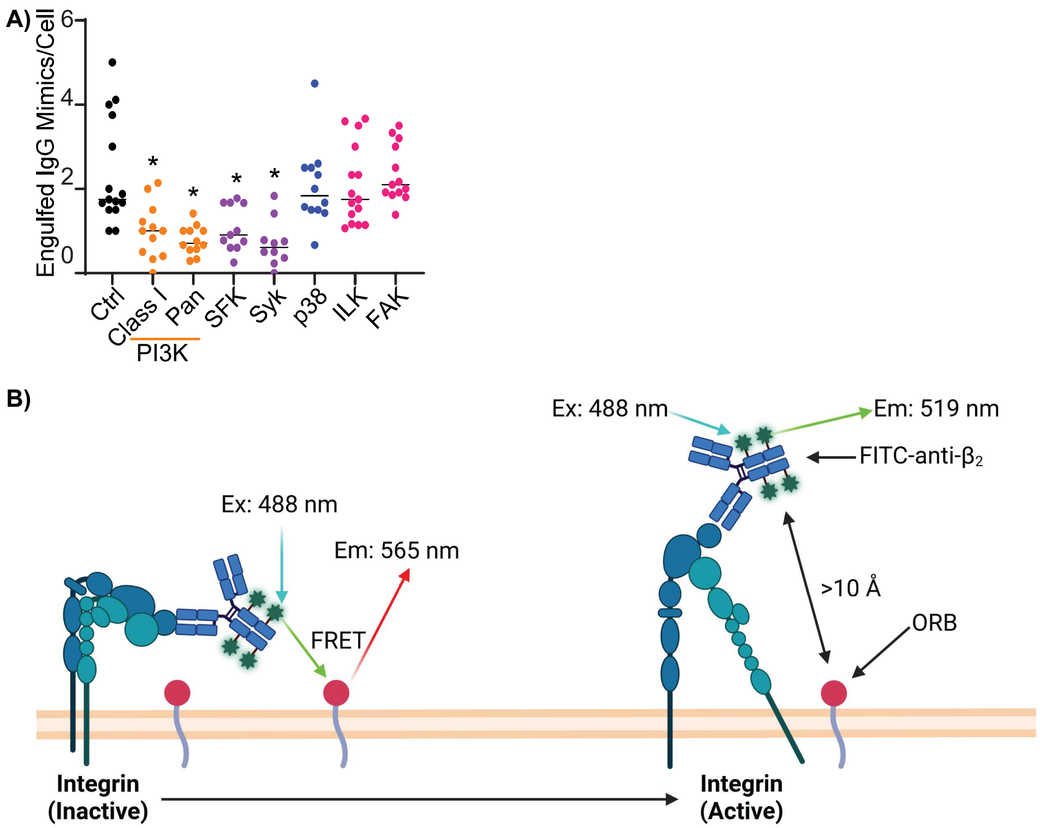


**Fig. S3. A)** Engulfment of IgG-oponized phagocytic targets by THP-1 macrophages treated with inhibitors of Class I PI3K (LY294002), Pan-PI3K (Wortmanin), SFK's (PP1), Syk (Syk-I), p38 MAPK (SB203580), ILK (Cpd-22), FAK (PF-00562271), or a vehicle control (Ctrl). **B)** Model of FRET-based integrin activation assay. When the β2 integrin is in the inactive (bent) conformation, the FITC-labeled anti-$\beta_2$ head-group-binding antibody TS1/18 will be positioned near the plasma membrane, where the excitation energy of FITC can be transferred to octadecylrhodamine B (ORB) embedded in the macrophage membrane. This results in the presence of ORB emission (565 nm) when FITC is excited using 488 nm excitation. Activation of the integrin causes a conformational change which will move the headgroup away from the plasma mem-brane, thereby positioning the TS1/18 antibody beyond the 10Å limit of FRET energy transfer. This causes a loss of the ORB fluorescent signal, and a concordant increase in the FITC emission at 519 nm.

Journal of Cell Science • Supplementary information

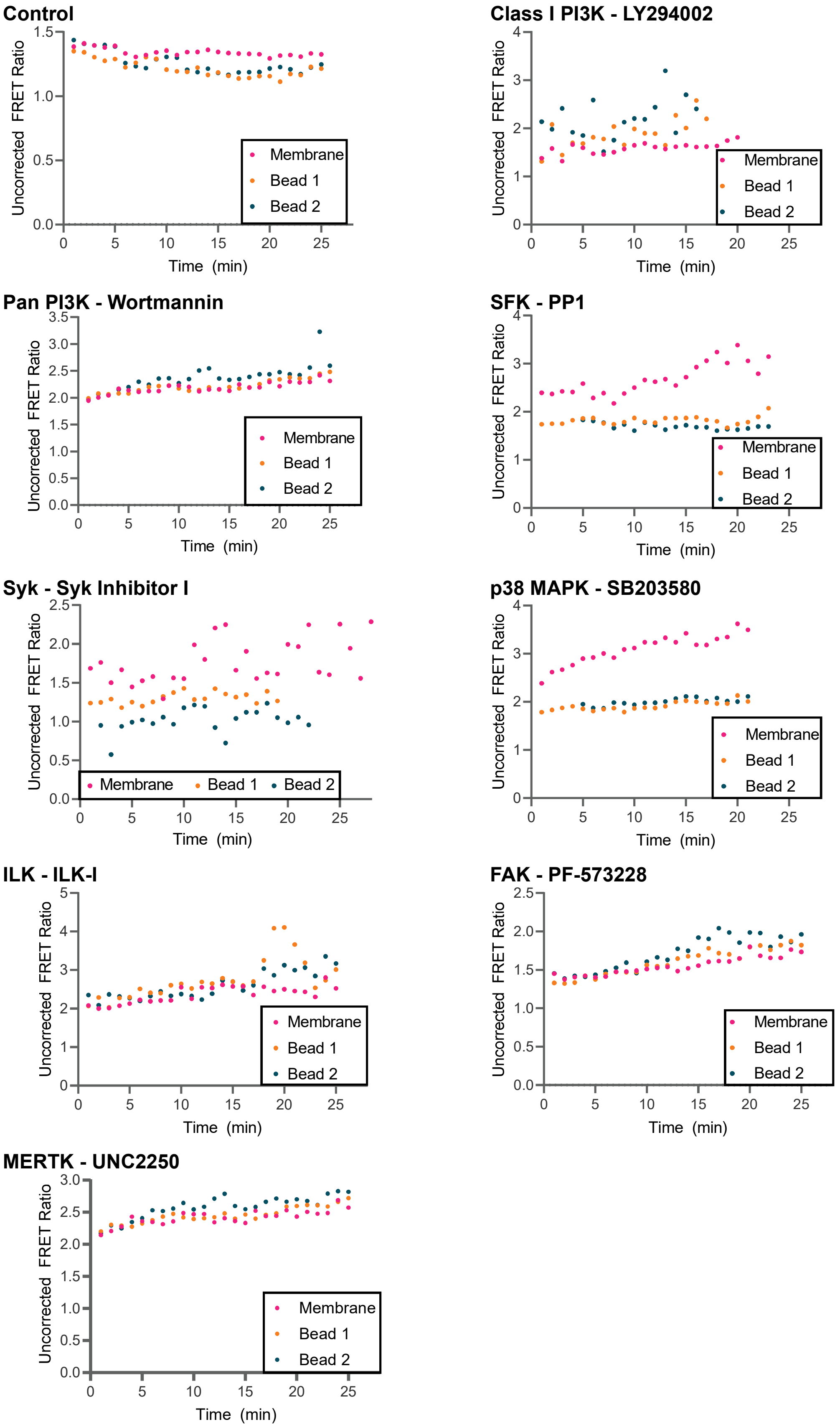


Journal of Cell Science • Supplementary information

**Fig. S4.** $\beta_2$ integrin activation dynamics, comparing the uncorrected FRET ratio between FITC and ORB of the bulk membrane (Membrane) versus the membrane in-contact with two representative Gas6-opsonized apoptotic cell mimics (Bead 1/Bead 2). $\beta_2$ integrin activation within the phagocytic cup is apparent as a decrease in the FRET ratio of the membrane contacting the beads relative to the bulk membrane. Cells are pretreated with the indicated inhibitors or a vehicle control. Data is representative of a minimum of 30 cells imaged in three separate experiments.

Journal of Cell Science • Supplementary information

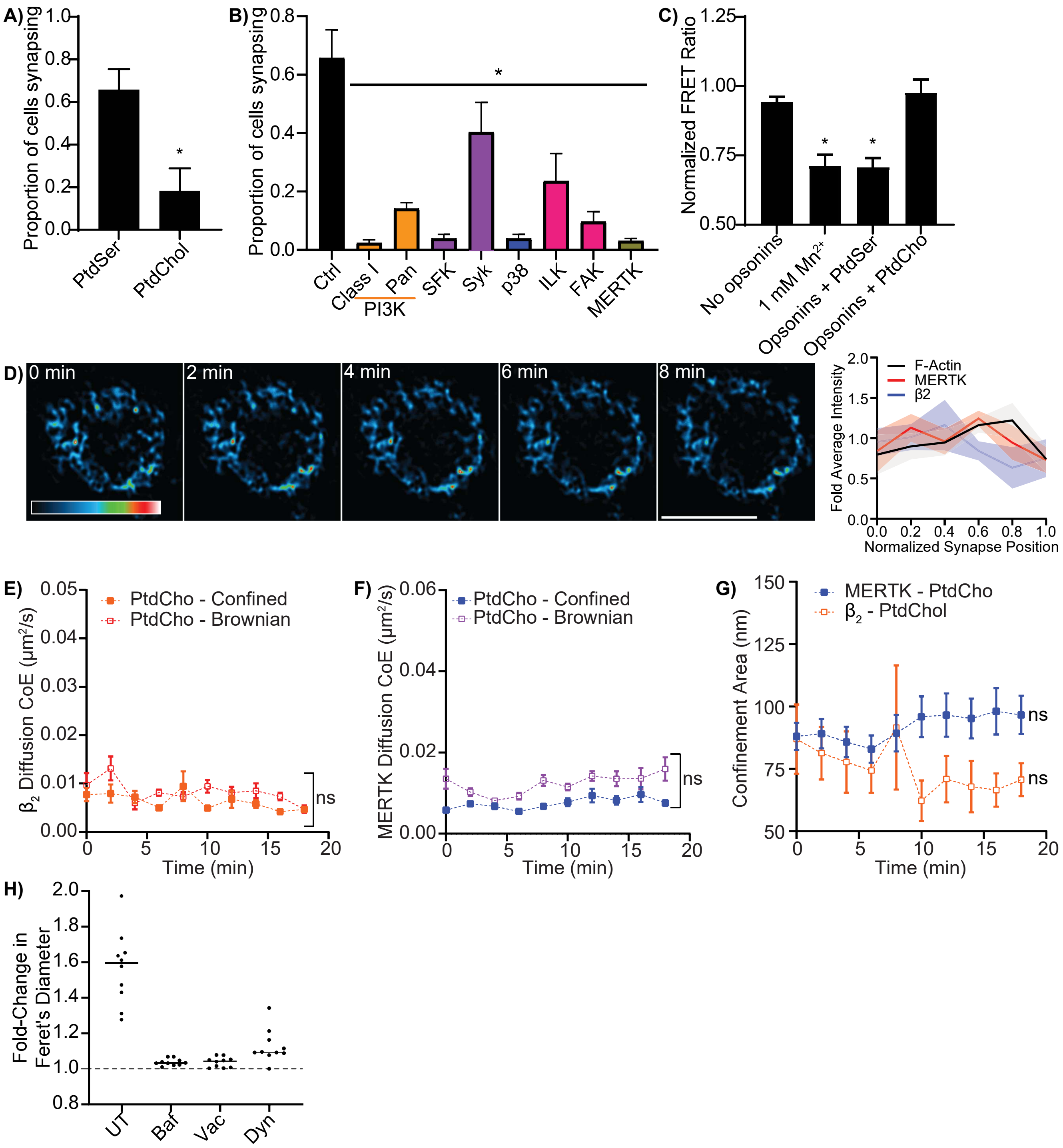


Journal of Cell Science • Supplementary information

**Fig. 5. A)** Portion of macrophages forming a synapse on Gas6 and MFG-E8 opsonized planar lipid bilayers containing either 20:80 PtdSer:PtdChol (PtdSer) versus 100% PtdChol. **B)** Effect of PI3K (LY & Wort), SFK (PP1 & SykI), p38 MAPK (SB), ILK (ILK-I), FAK (PF) and MERTK (UNC) inhibition, or a veihicle control (Ctrl) on the portion of THP1 macrophages which form a synapse when in contact with a planar apoptotic cell mimic. **C)** Activation of $\beta_2$ integrins, as quantified by FRET microscopy, on planar apoptotic mimics containing 20:80 PtdSer:PtdChol lacking opsonins (no opsonins), lacking opsonins and stimulated with manganese, opsonized with Gas6 and MFG-E8, or on a mimic containing 100% PtdChol that is opsonized with Gas6 and MFG-E8 (Opsonins + PtdCho). **D)** Image of actin distribution (left) and quantification of actin, MERTK, and $\beta_2$ integrin distribution in a macrophage interacting with a 100% PtdChol containing lipid bilayer. **E-F)** Diffusion coefficient of (E) $\beta_2$ integrin and (F) MERTK on a 100% PtdChol containing lipid bilayer. **G)** Confinement area size of MERTK and $\beta_2$ integrin on macrophages interacting with a 100% PtdChol containing lipid bilayer. **H)** Impact of inhibitors of exocyotiss (Bafilomycin [Baf] and Vacuolin-1 [Vac]) and endocyotsis (Dynasore [Dyn]) on synapse formation. Data are plotted as mean ± SEM, n = 3 to 5. * $p < 0.05$ compared to PtdSer (A), UT (B), no opsonins (C), or T=0 (E-G), Students T-test (A) or Kruskal-Wallis test with Dunn's multiple comparisons test.

Journal of Cell Science • Supplementary information

## Table S1. Antibodies and Other Staines Used in This Study.

| Target | Clone | Species & Isotype | Company | Catalog | Concentration* |
|---|---|---|---|---|---|
| FLAG | 6F7 | Rat IgG2 | Sigma-Aldrich | SAB4200119 | IB: 0.5 µg/mL<br>IP: 1.0 µg/mL |
| GAPDH | D16H11 | Rabbit IgG | Cell signaling | 5174 | IB: 1 µg/mL |
| α-tubulin | 236-10501 | Mouse IgG1 | ThermoFisher | A11126 | IB: 0.5 µg/mL |
| MERTK | Polyclonal | Rabbit IgG | Sigma-Aldrich | HPA075622 | IF: 0.5 µg/mL |
| Integrin $\beta_2$/CD18 | TS1/18 | Mouse | ThermoFisher | MA1810 | IF: 0.5 µg/mL |
| Integrin $\alpha_x$/CD11c | BU15 | Mouse | Biolegend | 337207 | IF: 0.5 µg/mL |
| FAK | EP695Y | Rabbit | Abcam | ab40794 | IF: 0.5 µg/mL |
| ILK | EPR1592 | Rabbit | Abcam | ab76468 | IF: 0.5 µg/mL |
| Syk | EP573Y | Rabbit | Abcam | ab40781 | IF: 0.5 µg/mL |
| Lyn | LYN-01 | Mouse | Abcam | ab1890 | IF: 0.5 µg/mL |
| p-AKT | polyclonal | Rabbit | Abcam | ab38449 | IF: 1.0 µg/mL |
| p-FAK | EP2160Y | Rabbit | Abcam | ab81298 | IF: 0.5 µg/mL |
| p-SFK | Y232 | Rabbit | Abcam | ab32078 | IF: 0.5 µg/mL |
| p-ILK | Polyclonal | Rabbit | ThermoFisher | PA5-12917 | IF: 0.5 µg/mL |
| p-SYK | EP573Y | Rabbit | Abcam | ab40781 | IF: 0.5 µg/mL |
| p-MERTK | Polyclonal | Rabbit | Abcam | ab14921 | IF: 0.5 µg/mL |
| Cy3 Conjugated Secondary Fab | Polyclonal | Goat | Jackson Immunoresearch | Various | IF: 0.1 µg/mL |
| DAPI | -- | -- | ThermoFisher | D1306 | IF: 0.5 µg/mL |
| Hoechst 33342 | -- | -- | ThermoFisher | 62249 | IF: 1 µg/mL |
| Wheat Germ Agglutinin-Alexa Fluor 647 | -- | -- | ThermoFisher | W32466 | IF: 5 µg/mL |

** Concentrations used for flow cytometry (FC), immunofluorescence (IF), immunoblotting (IB), or immunoprecipitation (IP).*

Journal of Cell Science • Supplementary information

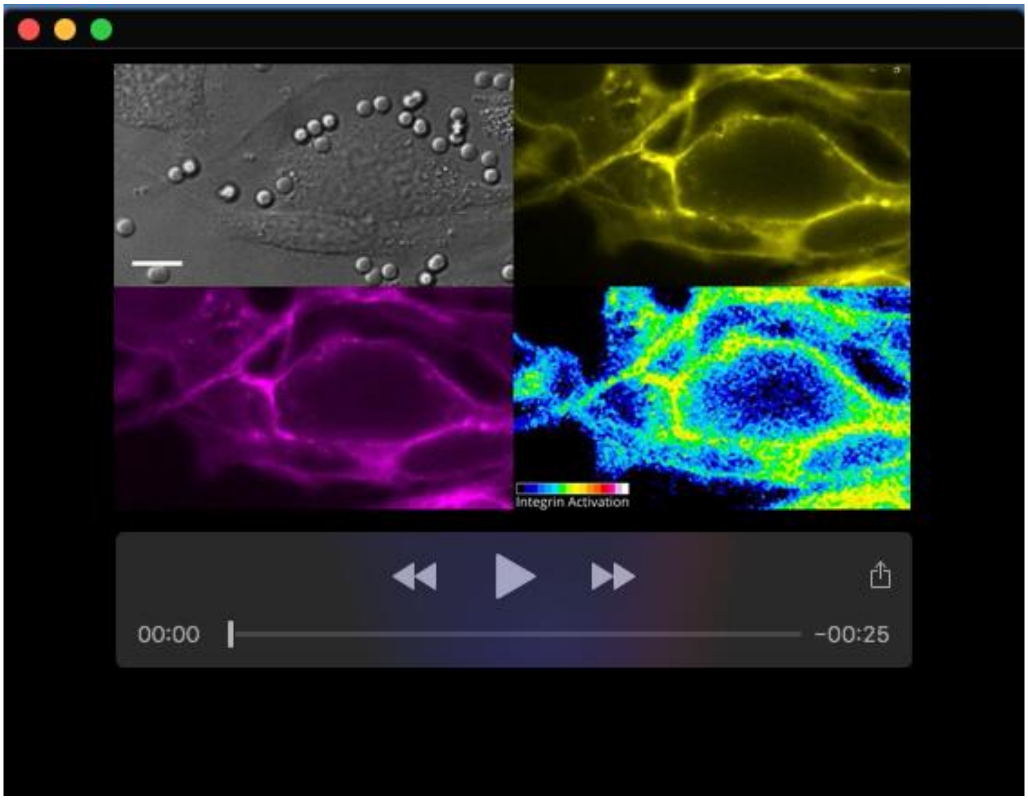


**Movie 1.** Timelapse of $\beta_2$ integrin activation on macrophages interacting with apoptotic cell mimics opsonized with the MERTK opsonin Gas6. Top-left shows a DIC image, yellow is the donor fluorophore attached to β2 integrin, magenta is the acceptor Octadecyl Rhodamine B. Heatmap shows the inverted FRET signal quantifying β2 integrin activation. Scale bar is 10 μm, 1 frame per minute.

Journal of Cell Science • Supplementary information

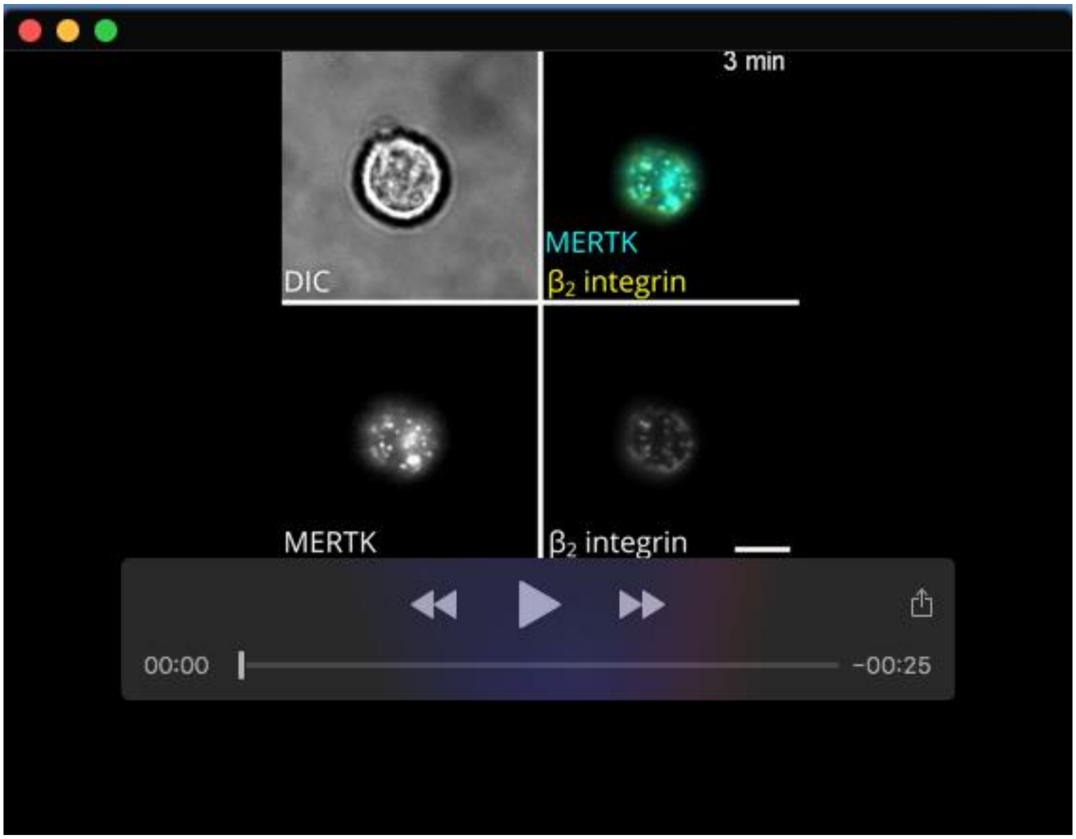


**Movie 2.** Efferocytic synapse formation of a THP-1 macrophage spreading on a supported lipid bilayer comprised of 20% phosphatidylserine and 80% phosphatidylcholine and opsonized with Gas6 and MFG-E8. Scale bar is 10 μm, 1 frame per minute.

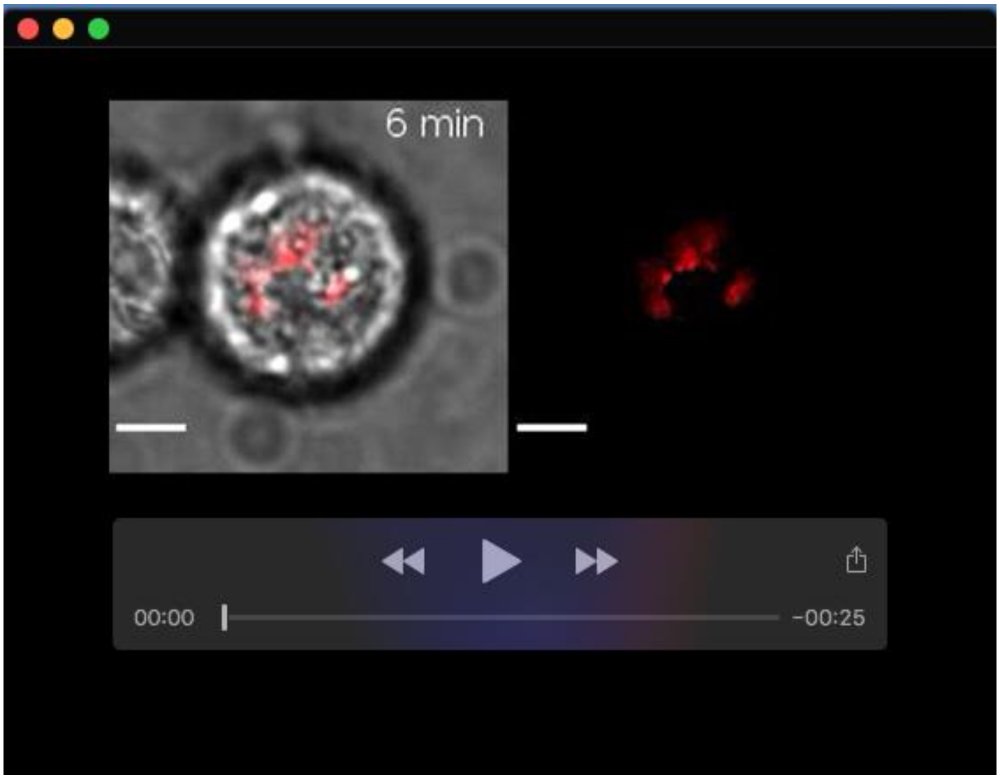


**Movie 3.** Localization of actin, stained with SiR-Actin, in a THP1 macrophage undergoing efferocytic synapse formation on a supported lipid bilayer comprised of 20% phosphatidylserine and 80% phosphatidylcholine and opsonized with Gas6 and MFG-E8. Scale bar is 10 μm, 1 frame per minute.

Journal of Cell Science • Supplementary information

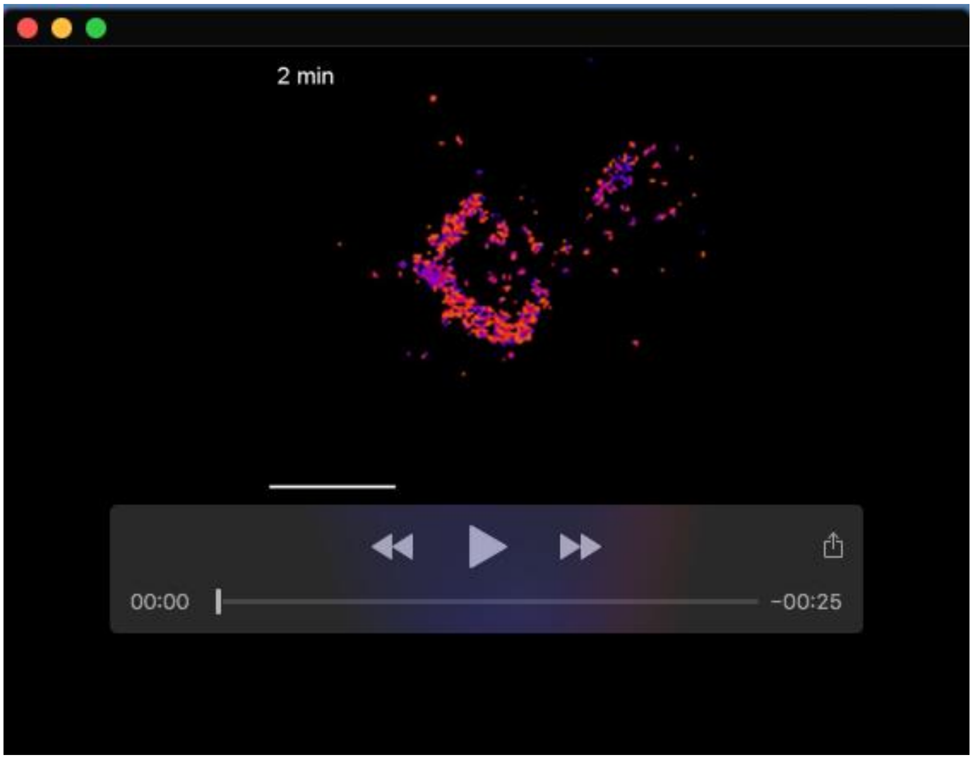


**Movie 4.** Timelapse of $\beta_2$ integrin activation, quantified by FRET between an integrin headgroup-recognizing antibody labeled with FITC and in the plasma membrane and Octadecyl Rhodamine B in the plasma membrane. The macrophage is undergoing efferocytic synapse formation on a supported lipid bilayer comprised of 20% phosphatidylserine and 80% phosphatidylcholine and opsonized with Gas6 and MFG-E8. Scale bar is 10 µm, 1 frame per minute.

Journal of Cell Science • Supplementary information

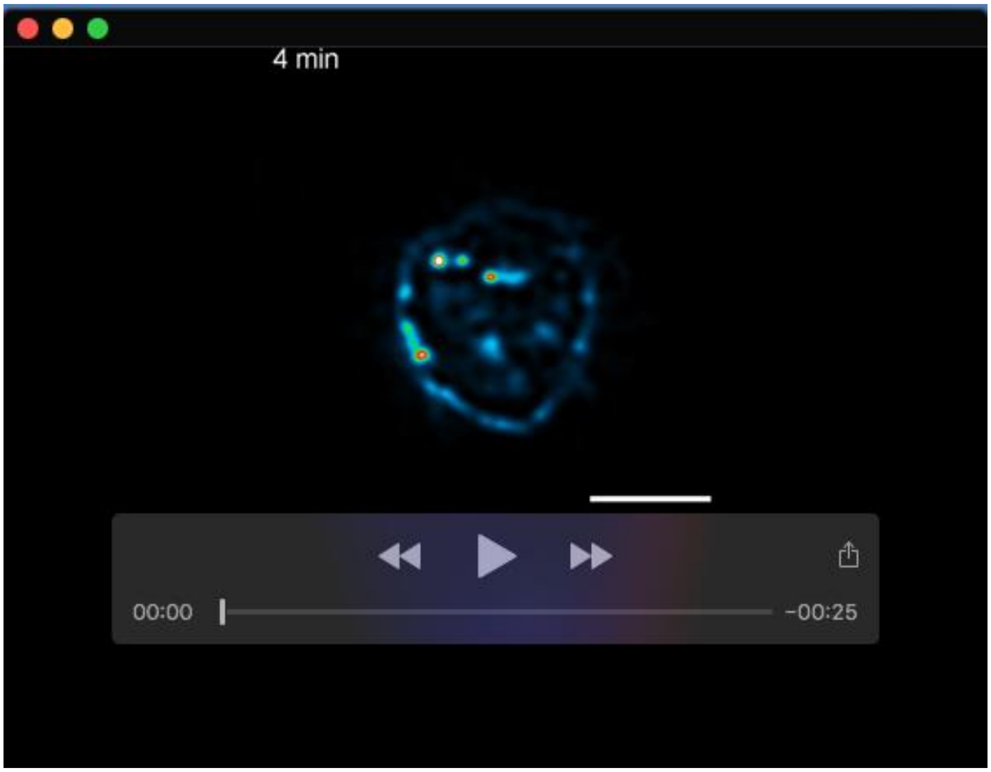


**Movie 5.** SRRF imaging of actin, stained with SiR-Actin, in a THP1 macrophage undergoing efferocytic synapse formation on a supported lipid bilayer comprised of 20% phosphatidylserine and 80% phosphatidylcholine and opsonized with Gas6 and MFG-E8. Scale bar is 10 µm, 1 frame per minute.

Journal of Cell Science • Supplementary information

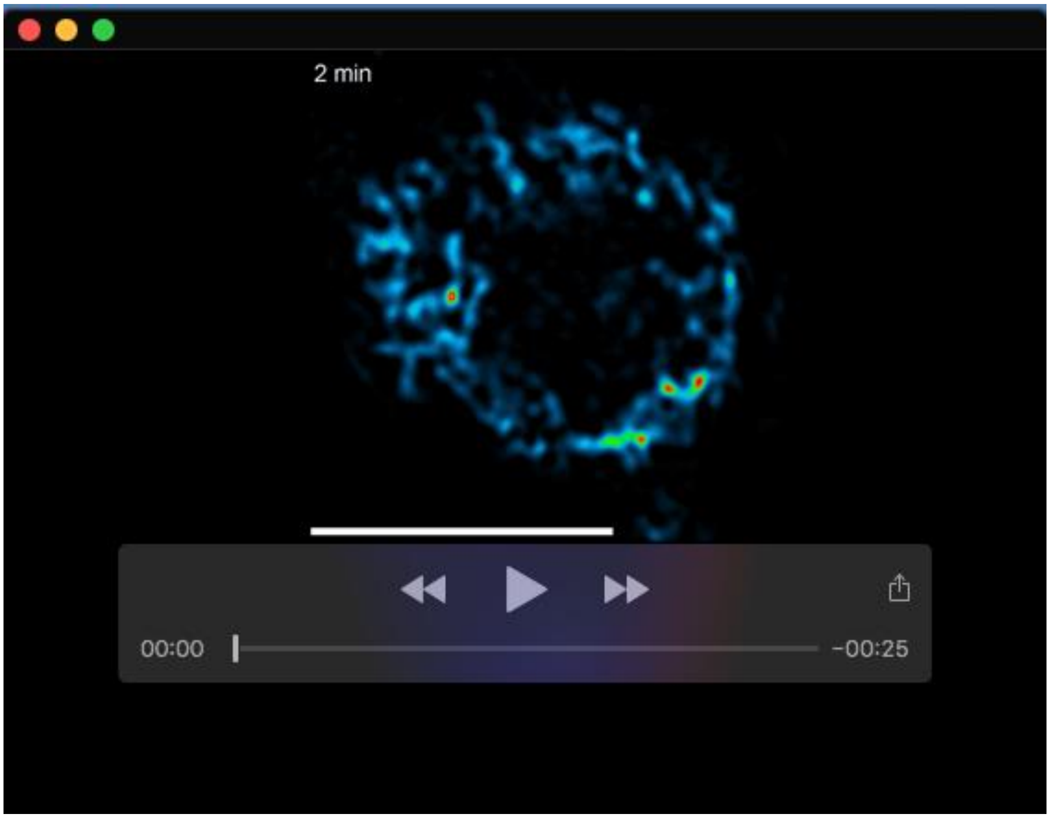


**Movie 6.** SRRF imaging of actin, stained with SiR-Actin, in a THP1 macrophage undergoing efferocytic synapse formation on a supported lipid bilayer comprised of 100% phosphatidylcholine and opsonized with Gas6 and MFG-E8. Scale bar is 10 µm, 1 frame per minute.

Journal of Cell Science • Supplementary information
